# Engineering MXene/metal composites from MAX phase/metal–Al precursors for high-performance energy conversion and storage

**DOI:** 10.1039/d5ra07113e

**Published:** 2025-11-10

**Authors:** Sergii A. Sergiienko, Andrei V. Kovalevsky, Jan Luxa, Kseniia Mosina, Bing Wu, Gabriel Constantinescu, Jalal Azadmanjiri, Nataliya D. Shcherban, Olena Diyuk, Zdenek Sofer

**Affiliations:** a University of Chemistry and Technology Prague Technická 5 166 28 Prague 6 Czech Republic sergiiee@vscht.cz sergiienko@ua.pt; b Department of Materials and Ceramics Engineering, CICECO – Aveiro Institute of Materials, University of Aveiro 3810-193 Aveiro Portugal; c L.V. Pisarzhevsky Institute of Physical Chemistry of NAS of Ukraine 31 Nauki Ave. Kyiv 03028 Ukraine; d Environmental Chemistry Group Institute of Physical Chemistry PAS Marcina Kasprzaka 44/52 01-224 Warszawa Poland; e TEMA – Centre for Mechanical Technology and Automation, Department of Mechanical Engineering, University of Aveiro 3810-193 Aveiro Portugal

## Abstract

Two-dimensional (2D) transition metal carbides, MXenes, have garnered significant interest for their potential applications in various energy storage and conversion systems. This work presents a novel approach to synthesizing MXene/metal composites *via* direct etching of MAX phase/metal–Al alloy precursor materials. By combining the Ti_3_AlC_2_ MAX phase with metal alloys and optimizing the processing conditions, we demonstrate a scalable method for producing MXene/metal composites with a 3D porous architecture and good mechanical integrity. Electrochemical tests have revealed that these composites exhibit high electrochemical activity toward the hydrogen evolution reaction (HER) and possess substantial areal capacitance, making them promising candidates for energy storage applications. Additionally, this synthesis strategy is adaptable to other MAX phases and metal–Al alloys, enabling the fabrication of customizable MXene/metal composites with tailored nanostructures for a broad range of electrochemical applications.

## Abstract 2

The MXene/metal composites with a 3D porous structure prepared in this work were evaluated for energy conversion and storage applications, using the hydrogen evolution reaction under alkaline conditions as a model system.

## Graphical abstract

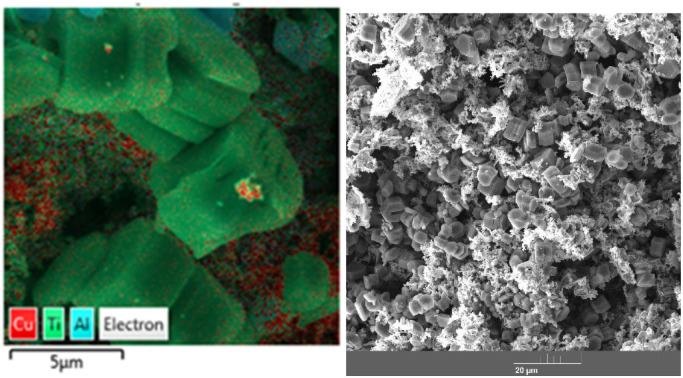

## 1 Introduction

The exploitation of renewable wind and solar energy for sustainable development is an important part of national and international energy policies. Due to the variability of wind and solar power, energy storage systems that allow effective management of power generation are required.^1^ Today, several technologies are considered for energy storage, among which hydrogen production by water electrolysis is regarded as one of the most promising approaches. Ammonia is considered a potential hydrogen carrier, however, the NH_3_ electrosynthesis from N_2_ and H_2_O looks attractive, but is currently limited by its low efficiency.^2^ Electrochemical CO_2_ reduction is another attractive technology for the production of liquid fuels.^3^ All these technologies require efficient and cost-effective electrocatalysts for successful industrial implementation. MXene/metal composites are regarded as promising electrocatalysts for these processes.^4^

Ni and Ni-based alloy (Ni–Cu) electrodes have been employed as non-precious metal catalysts for hydrogen production owing to their substantial catalytic activity toward the hydrogen evolution reaction (HER) in alkaline media.^5^ However, in low-cost alkaline water electrolysers, the sluggish water dissociation kinetics (Volmer step) of platinum-free electrocatalysts results in poor hydrogen production activity. As HER electrocatalysts, MXene/nano-Ni composites demonstrate a lower overpotential in alkaline media compared with bulk Ni electrode. An enhanced catalytic activity of MXene/nano-Ni composite is attributed to the acceleration of the Volmer step in HER process in alkaline media, by enhancing the water adsorption and dissociation on the catalyst surface, which is a rate-limiting step.^6,7^ Bulk copper is a poor electrocatalyst for HER. This property has been exploited to develop an MXene/nano-Cu catalyst with high selectivity for the nitrogen reduction reaction (NRR) and NH_3_ electrosynthesis, where selecting a metal support with low HER activity is crucial for MXene-based catalysts.^2^

Cu-based electrocatalysts are also highly promising electrocatalysts for the carbon dioxide reduction reaction (CO_2_RR). Combining Cu with MXene leads to a significant enhancement in the CO_2_RR performance.^8^ Other possible directions for the application of MXene/metal composites are metal-ion batteries and supercapacitors.^9,10^

High electrical conductivity is crucial for bulk MXene-based electrodes to ensure optimal performance. Thick MXene-based multi-layered films generally have lower electrical conductivity (7000 S cm^−1^ for ∼23 μm,^11^ 3000 S cm^−1^ for ∼5 μm)^12^ compared to thinner films (15 100 S cm^−1^ for ∼200 nm)^13^ or a single monolayer Ti_3_C_2_T_*x*_ flake (24 000 S cm^−1^ for 1 nm)^14^ due to a greater number of inter-flake interfaces.^15^ However, it is well known that, as the electrode thickness decreases, the areal capacitance and electrocatalytic activity tend to decline, limiting potential practical applications.^16^

Similar to other 2D materials, MXene nanosheets tend to stack and aggregate due to strong van der Waals forces. Therefore, owing to the volume swelling and weak conductivity, long-term stability and high-rate capacity of MXene materials have not been achieved. It is assumed that the MXene-based composites with a 3D porous structure are more stable and less susceptible to restacking problems caused by van der Waals forces and hydrogen bonding between the 2D nanosheets.^9,17,18^ Different additives are used to improve areal capacitance and mechanical stability of thick multi-layered MXene-based electrodes, such as polymer binders (*e.g.*, PVDF), and carbon black for increasing interflake electrical conductivity.^19^ MXene/metal composites exhibit more metallic behavior than ceramic compared to pure MXenes offering potential applications in areas such as electrocatalysis and energy storage.^20^ Despite the corrosion of metal current collectors in water-based electrolytes being one of the main factors limiting the use of metal current collectors,^21^ the potential to improve MXene-based electrodes through the formation of such composites appears attractive. In such MXene/nano-metal composites, the nano-metal acts as a binder, internal current collector at the interflake interfaces, and co-catalyst.

Nanoporous metals are known to possess high surface area, high electrical conductivity (for copper, 5.95 × 10^5^ S cm^−1^) and mechanical characteristics.^22^ Nanoporous noble metals themselves usually have low specific capacitance, *e.g.* nanoporous gold exhibits capacitance values of up to 10 F g^−1^. The relatively low values are often attributed to the limited electrochemical activity of gold.

Nanoporous transition metals attract attention for practical applications due to their rich redox chemistry. The combination of porous metals with pseudocapacitive materials (*e.g.*, transition metal oxides, MXenes) has proven effective in improving capacitive performance.^23^ Among different transition metals, nickel-based electrodes have been considered among the most promising materials, since they possess higher theoretical specific capacitance, are environmentally friendly and cost-effective. However, hierarchical nanoporous non-noble metals have not yet been realized. This is mainly owing to the poor pore-forming ability and low chemical stability of transition metals.^24^ Still, some recent studies of porous Ni–Cu alloys have reported high redox capacitance values (100–600 F g^−1^) and good stability in alkaline electrolytes.^25^

In electrocatalysts, supercapacitors, and batteries the porous structure is required for rapid ion diffusion. Thus, the electrodes should preferably have large pores (50–100 nm) to facilitate electrolyte diffusion and small pores to ensure a large internal surface area.^24,26^ After the etching of Al from the parent MAX phase followed by delamination, MXene multilayered crystals can create nanopores between the Ti_3_C_2_ layers. As a result, larger pores may form in the metal phase of MXene/metal composites, enhancing their structural and functional properties.

Large porous structures in MXene/metal composites can be formed during the synthesis stage of MAX phase/metal composites by methods similar to those used for producing porous metals. One such method is space-holder casting using inorganic granules or soluble salts. The space-holder granules are then removed by leaching in suitable solvents.^27^ A key advantage of using space holders is the ability to control the pore size distribution, which is determined by the particle size distribution of the filler granules.

Most MXene/metal composites are obtained by the reduction of metal salts in solution, with subsequent deposition of metal nanoparticles (NPs) on the surface of MXene flakes for the development of highly conductive materials.^28–30^ Metal NPs can enhance local electrical pathways especially, when metal loading is sufficiently high or when the metal forms interconnected networks, resulting in a noticeable increase in overall electronic conductivity compared with bare MXene. If NPs are isolated and not well connected, the gains in conductivity are modest. However, the functional groups at the surface of MXene hinder the direct formation of such composites, leading to poor wettability between MXene and metals.^31^ Although such composites exhibit higher conductivity values (27 193 S cm^−1^ for MXene/Ag nanowire composite^32^ or 1.6–2.7 times higher than that of Ti_3_C_2_T_*x*_ for MXene/metal NPs composite^33,34^) this is still insufficient for the formation of thick films. Higher NPs loadings can improve catalytic activity and electronic conductivity, but too high a loading may lead to NP aggregation and restacking of MXene.^35^ To date, it remains challenging to improve the mechanical properties of MXene film without largely affecting its superb electrical conductivity.^36^ The use of polymer binders can moderately reinforce MXene films but sharply decreases their electrical conductivity.^37^

An alternative approach enables the direct synthesis of MXene/metal (Ti_3_C_2_(OH)_*x*_/nano-Ni) composites by etching MAX phase/metal–Al alloy composites (Ti_3_AlC_2_/Ni_2_Al_3_) under alkaline conditions.^38,39^ Many previous attempts to obtain MAX phase/metal composites or to introduce metallic dopants into the MAX phase during sintering were unsuccessful because the Al–metal bond energy in alloys (*e.g.*, Ni_3_Al) is higher than in MAX phases (*e.g.*, Ti_3_AlC_2_). This leads to MAX phase decomposition and preferential formation of conventional carbides (TiC) and Ni–Al alloys, particularly at low Al content.^40^ The possibility of simultaneous synthesis or sintering of MAX phase/metal–Al alloy (Ti_3_AlC_2_/Ni_2_Al_3_) composites from elemental precursors is possible when the reaction mixture contains an excess of Al, and metal additive forms an Al-saturated phase (Ni_2_Al_3_ or NiAl_3_) with a lower Al–metal binding energy, thereby preventing MAX phase decomposition.^38^

Promising candidate metals for A-element–metal alloys may be those that form alloys with the A element (Al) but do not form MAX phases. These metals include Ni, Cu and some others, and their corresponding alloys (Cu–Al, Ni–Al) are often used as precursors for the synthesis of nanoporous metals by dealloying.^26,41,42^ Moreover, monolithic samples of these nanoporous metals can be fabricated through chemical dealloying of corresponding alloys Al–Cu,^43^ Al–Ni–Cu,^44^ Ni–Cu–Mn,^24^ and Ni–Cu–Al–Mg.^45^

Hence, MAX phase/Ni–Al alloy composites can be used as precursors for the synthesis of MAX phase/MXene/Ni composites by direct Al etching and dealloying under alkaline conditions.^38,39^ However, the MXene content in such composites is relatively low due to incomplete Al etching. Etching of Al from Ti_3_AlC_2_/Ni_2_Al_3_ composites in fluorine-containing media may also cause Ni dissolution.^38,39^

Among various metals, those that are stable in fluorine containing media or form stable alloys are of particular interest.^46^ These metals include noble metals and Cu. The standard electrode potential of Cu^2+^/Cu is +0.342 V *versus* SHE, which facilitates the dealloying Al from Cu–Al alloys and the formation of nanoporous metallic structure.^47^ Another interesting material is sintered porous Ni–Cu alloys^48^ that exhibit superior corrosion resistance in aqueous hydrofluoric acid compared to porous Ni.^49^ Thus, it can be assumed that MXene/metal porous composites can be obtained by selective Al etching of bulk sintered MAX phase/Cu–Al or Ni–Cu–Al alloy composites in HF or alkaline solutions.

Therefore, this work aims to advance this field by developing electrode concepts for electrochemical energy conversion and storage systems, enabled by a facile synthesis of MXene/nano-metal composites (metal: Ni, Cu; MXene – Ti_3_C_2_T_*x*_ (T = F, OH)) with a 3D porous structure *via* etching of MAX phase/metal–Al alloy precursors.

## 2 Materials and methods

### 2.1 Initial precursors

The samples were synthesized from commercially available powders of titanium (−325 mesh, less than 45 microns, 99%), carbon black acetylene (50% compressed, 99.9+%), nickel (APS 3–7 micron, 99.9%), aluminium (−325 mesh, 99.5%, metal basis, APS 7–15 microns), and copper powder (−325 mesh, 99% metals basis). Before mixing with other components, carbon black was subjected by ball milling for 8 hours (2 g of carbon black and 30 ml of ethanol) to decrease particle size, and then dried at 60 °C degrees for 2 hours.

### 2.2 Preparation of MAX phase/metal–Al alloy composites

The initial powders (Ni, Cu, Ti, Al, C) were sequentially mixed with ethanol to obtain a suitable homogeneous slurry and then dried at 60 °C for 2 hours. Next, 4 g of the powder mixture were pressed (at 10 kN) uniaxially in the shape of discs (25 mm in diameter and ∼3 mm thick). The samples were placed on top of an Al_2_O_3_ powder layer in an alumina crucible and were also covered with an Al_2_O_3_ powder layer to reduce the evaporation of Al. To determine the optimum sintering conditions the powder compacts were sintered to form MAX phase/metal–Al alloy (metal – Cu or Ni) composites using conventional sintering (CS) of the pressed powders in an Ar atmosphere at different temperatures (800–1450 °C) and time (from 1 minute to 4 hours, with a heating/cooling rate of 5 °C per minute) (Table S1). Further, samples sintered at 1200 °C for 1 hour were used for research. Initial compositions, corresponding denominations and processing parameters of the samples are listed in Table 1.

**Table 1:** Initial composition and denominations of the samples sintered at 1200 °C for 1 hour

Sample denomination after sintering	Initial composition, molar ratios					Sample denomination after etching in HF
	Ni	Cu	Ti	Al	C	
1			2	4.5	1	1b
2	0.5		2	4.5	1	2b
3	1		2	5	1	3b
4	0.25	0.25	2	4.5	1	4b
5	0.35	0.35	2	4.5	1	5b
6	0.75	0.75	2	4.5	1	6b
7		0.5	2	4.5	1	7b
8		1	2	4.5	1	8b
9		1.5	2	4.5	1	9b
10		3	2	7.2	1	10b

### 2.3 Preparation of MXene/nano-metal composites

The MXene/nano-Cu and MXene/(NiCu)_2_Al_3_ composites were prepared both in the form of powders and as bulk samples (layers on the surface of sintered MAX phase/metal–Al alloy samples) by direct etching in acid solution (Table 1).

### 2.4 Preparation of MXene/nano-metal composites (powders)

MXene/(NiCu)_2_Al_3_ sample 4b was prepared by etching of MAX phase/(NiCu)_2_Al_3_ composite sample 4 (4 g) only in HF solution. Two stages of etching in HF solution (20 wt% in water) were applied, with the first one in 14 ml for 1 day. Second stage of etching in fresh HF solution (14 ml) for 1 day was used to remove Al from residual MAX phase. Molar Al : HF ratio was approximately equal to 1 : 8, considering the total amount of Al in the MAX phase. (NiCu)_2_Al_3_ phase reacts much more slowly with HF acid, probably due to the formation of a surface layer consisting of a corrosion-resistant Ni–Cu alloy.

MXene/nano-Cu samples 7b, 8b, 9b, 10b, were prepared by etching MAX phase/Cu–Al samples 7, 8, 9, 10, respectively (Table 1). First, the samples were etched in an excess of HCl solution (17.5 wt% in water) for 1 day to form a porous MAX phase/Cu structure. Molar Al : HCl ratio was set to 1 : 9, considering the total amount of Al in the sintered samples. Next, two stages of etching in HF solution were applied. First stage included etching in HF solution (9 ml, 20 wt% in water) for 1 day. Next, a second etching in an excess of fresh HF solution (9 ml, 20 wt% in water) was applied. Al : HF molar ratio was equal approximately to 1 : 8, considering the total amount of Al in MAX phase.

### 2.5 Preparation of bulk MXene/nano-metal composites

MXene/(NiCu)_2_Al_3_ composite (bulk sample 5b) was designed as a layer on the surface of MAX phase/(NiCu)_2_Al_3_ sintered sample 5 (mass ∼4 g before etching, diameter ∼23 mm, thickness ∼3 mm, geometric electrode area ∼10 cm^2^), using direct etching in a limited amount of HF solution to prevent sample disintegration. As a first step, etching in HF solution (6 ml, 20 wt% in water) for 1 day was applied. Next, the etching was continued in a fresh HF solution (6 ml HF 20 wt% in water) to form MXene/(NiCu)_2_Al_3_ composites. The amount of HF was chosen to dissolve approximately half of the Al in the samples.

MXene/nano-Cu composite (bulk sample 8b) was formed as a surface layer on the MAX phase/Cu–Al bulk sample 8 (mass of ∼4 g before etching, diameter ∼22 mm, thickness ∼3.5 mm, geometric electrode area ∼10 cm^2^). This was achieved by direct etching in a limited amount of acid solution, while avoiding sample destruction. First, the porous MAX phase/Cu structure in the sample was produced by etching in HCl solution (15 ml, 17.5 wt% in water) for 1 day. The amount of HCl was chosen to dissolve approximately half of the Al in the sample and to prevent disintegration. In the second stage, etching in HF solution (8 ml, 20 wt% in water) was applied to form MXene/nano-Cu composite.

**Fig. 1:**
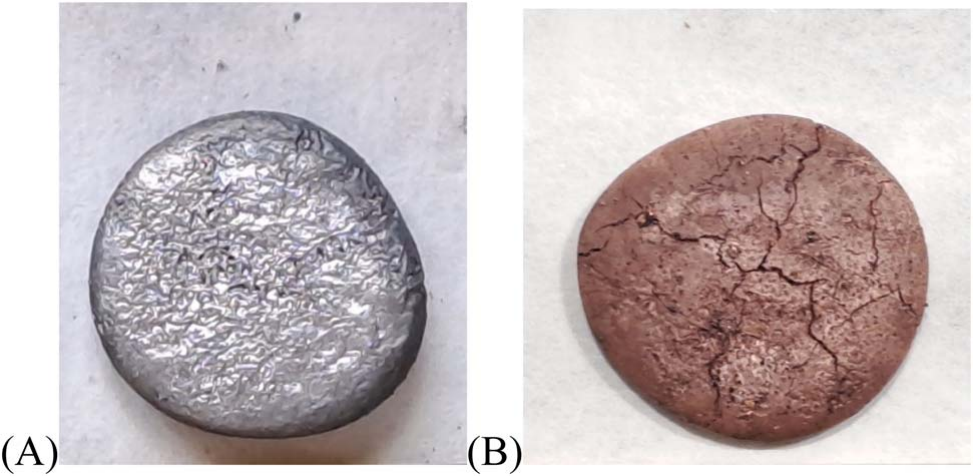
Images of the samples: MAX phase/CuAl_2_ sample 10 after sintering (A), MXene/Cu sample 10b after sintering and etching (B).

The MXene/nano-Cu composite (bulk sample 10b) with a 4 mm thick 3D porous structure was formed by completely etching Al from the MAX phase/Cu–Al bulk sample 10 (Fig. 1A). This sample had a mass ∼4 g before etching, diameter ∼17 mm, thickness ∼4 mm, geometric electrode area ∼6.7 cm^2^. First, the porous MAX phase/Cu structure in the sample was produced by etching in an excess of HCl solution (50 ml, 17.5 wt% in water) for 4 days. In the second stage, etching in an excess of HF solution (10 ml HF 20 wt% in water) was applied to form MXene/nano-Cu composite (Fig. 1B).

After each etching stage, all samples were washed with distilled water to remove AlCl_3_ or AlF_3_. For delamination, TBAOH solution (20 wt% in water) was used for 1 day. Next, the samples were washed with distilled water to dissolve the residual TBAOH. Samples in the form of powders were filtered through filter paper and dried at room temperature for 1 day.

### 2.6 Material characterization

The phase composition was assessed by X-ray diffraction analysis (XRD), using a Brucker D8 Advance diffractometer system (Cu Kα radiation, 0.154056 nm). In order to prepare powders from the initial sintered products for XRD analysis, the sintered products were ground to reduce the average particle size to below ∼50 μm.

Scanning Electron Microscopy (SEM, Tescan MAIA 3, Czech Republic) equipped with an Energy Dispersive Spectrometer (EDS, Oxford Instruments, England) was used to characterize the relevant microstructural features.

X-ray photoelectron spectroscopy was carried out on a Phoibos 100 analyzer (SPECS, Germany) using a monochromatic Al Kα1 source (1486.7 eV). The samples were placed onto conductive Cu tape. Samples analysed after HER activity measurements were measured on screen printed electrodes. For survey spectra, an analyzer pass energy (*E*_pass_) of 50 eV and a step size of 1 eV were used, while for high-resolution core-level spectra a pass energy of 20 eV with a step size of 0.1 eV was used. Charge compensation using a flood gun was unnecessary due to the good electrical conductivity of the samples.

### 2.7 Electrochemical measurements

Electrochemical measurements were carried out using an Autolab PGSTAT204 potentiostat connected to a three-electrode electrochemical cell. A Pt foil (2 cm^2^) was used as a counter electrode and Hg|HgO (1 M NaOH), +0.098 V *vs.* standard hydrogen electrode (SHE) as the reference electrode. Experiments were performed at room temperature (20 °C) with NaOH (1 M) as the electrolyte.

Two types of working electrodes were tested. As the first type of electrodes, copper foam (thickness ∼1 mm, porosity 98%, 110 pores per inch, which approximately corresponds to an average pore size of 200 μm) was used as substrate. Active material was prepared by mixing 0.2 g MXene-based composite with 0.08 ml PVDF solution in NMP (15 mg ml^−1^) together. The prepared slurry was deposited on copper foam on both sides (total geometric surface area ∼3.5 cm^2^) and dried at 35 °C for 48 h under vacuum. The mass loading of the active material was ∼130 mg per 3.5 cm^2^ or ∼37 mg cm^−2^.

The second type of working electrodes are bulk samples 5b, 8b and 10b, prepared by etching without destruction.

Hydrogen evolution tests were performed between −0.4 and −1.5 V with a scanning rate of 0.1 mV s^−1^. The current density values were calculated with respect to the geometric surface area of each electrode. The stability test was carried out in 1 M NaOH solution for samples 7b, 4b for 20 days at −1.1 V *vs.* Hg|HgO.

In order to estimate electrochemically active surface area (ECSA) of obtained samples, the double-layer capacitance method was used.^50^ The ECSAwas calculated from the double-layer capacitance (*C*_dl_) as: ECSA = *C*_dl_/*C*_s_. The specific capacitance values (*C*_s_) for a flat standard with 1 cm^2^ of the real surface area is generally in the range of 20 to 60 μF cm^−2^ (40 μF cm^−2^ was taken as the average value).^51^ To estimate *C*_dl_, cyclic voltammetry (CV) was used, and these data were recorded around the open circuit potential (OCP) using different scan rates (*V*_b_) from 1 to 100 mV s^−1^. Further, the double-layer capacitance was calculated as *C*_dl_ = Δ*J*/*V*_b_. The double-layer capacitance (*C*_dl_) for samples was estimated by plotting the Δ*J* = (*J*_a_ − *J*_c_)/2 (at the overpotential in the middle of the cycling range) against the CV scan rate, where *J*_a_ and *J*_c_ are the anodic and cathodic current densities, respectively. ECSA values were normalized to 1 cm^2^ of surface area (areal electrode density ∼37 mg cm^−2^), for balk samples (5b, 8b), areal density estimation was difficult, so the data are presented per unit geometric area (1 cm^2^). This seems to be a suitable approximation because the measured specific capacitance also accounts for pseudocapacitance in addition to electrical double-layer capacitance.^52,53^

The cyclic voltammograms (CV) and galvanostatic charge–discharge (GCD) curves were recorded in the potential window ranging from −0.4 to −0.9 V. The GCD profile of the samples were recorded at 0.08 A g^−1^ (2.8 mA cm^−2^). The values of areal and gravimetric capacitance (C1) were calculated from CV curves using the simplified formula *C* = Δ*J*/*V*_b_, and the values of Δ*J* were obtained near the open circuit potential (OCP) approximately at −0.75 V. As discussed below, the conventional method of capacitance calculation by current integration over time may not be suitable in this case.^54^ To facilitate direct comparison of the capacitive performance of different materials (areal capacitance), CV curves were plotted as specific capacitance (F cm^−2^) *versus* potential instead of current density. The areal capacitance values were calculated from current–voltage curves at each potential point using the formula mentioned above (*C* = Δ*J*/*V*_b_).

Electrochemical impedance spectroscopy (EIS) was carried out using the same potentiostat at the open circuit potential (−0.75 V), with a 50 mV signal amplitude and frequencies ranging from 20 kHz to 5 mHz.^55^

## 3 Results and discussions

### 3.1 General considerations on the formation and characterisation of MAX phase/Ni–Cu–Al composites

**Fig. 2:**
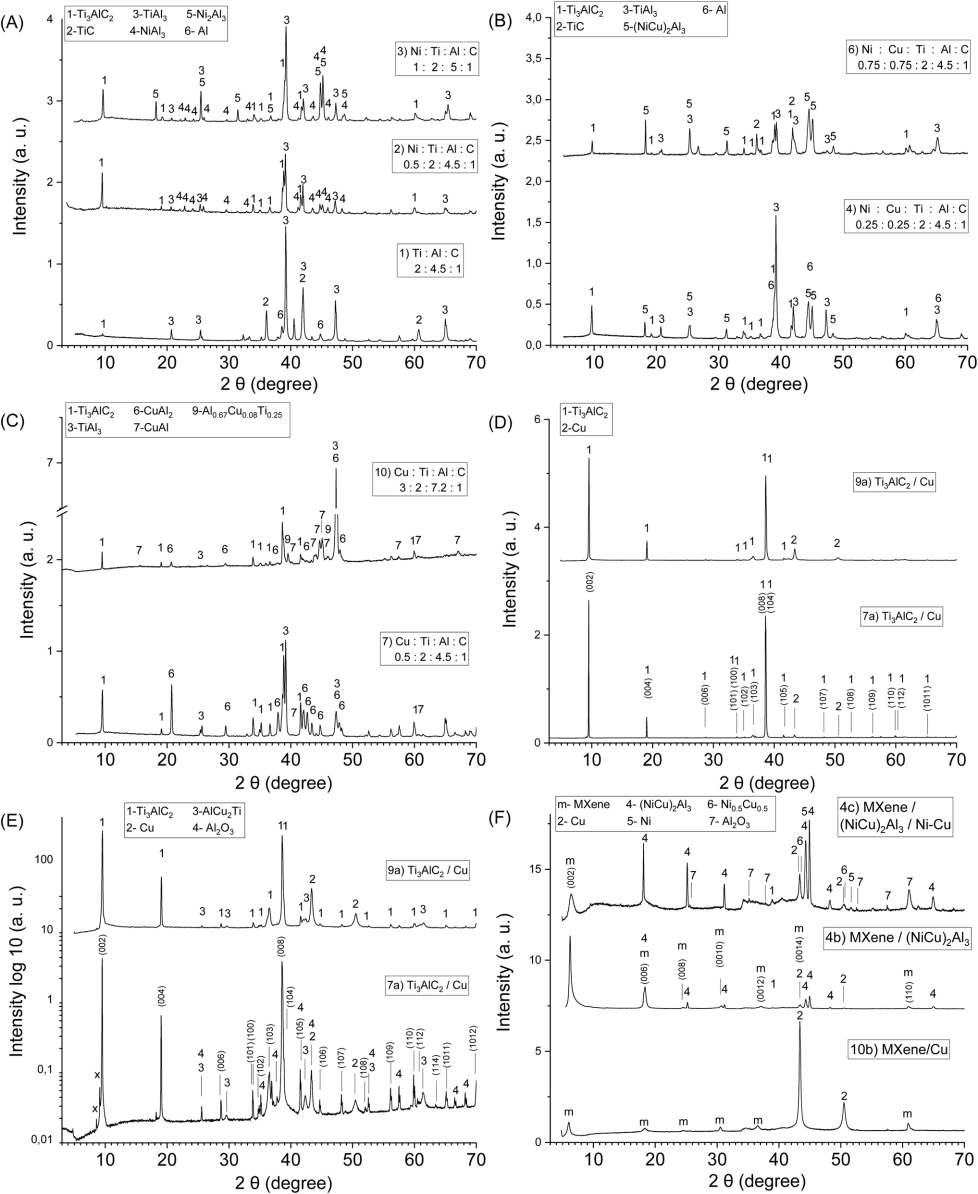
XRD data of the composites: sample 1 (without Ni or Cu addition), sample 2 (with NiAl_3_ alloy), sample 3 (with Ni_2_Al_3_ and NiAl_3_ alloys) (A); samples 4, 6 with different (NiCu)_2_Al_3_ alloys content, (B), sample 7, 10 with different Cu–Al alloys content (C), samples 7a, 9a (samples 7, 9 after etching in HCl solution) (D and E), sample 10b (MXene/Cu), sample 4b, c (MXene/(NiCu)_2_Al_3_) (F).

The presence of Ni–Al alloys (Ni_2_Al_3_, NiAl_3_) in the reaction mixture allows a reduction in the synthesis temperature and duration in the preparation of Mo_2_TiAlC_2_ MAX phases.^39^ In the literature, this technique is commonly referred to as liquid phase sintering (LPS).^56^ It can accelerate the diffusion of components, enhance mass transfer in the reaction mixture, and thereby promoting the formation of final products and reduce the content of intermediate compounds. However, etching Al from Mo_2_TiAlC_2_ MAX phase requires higher temperatures and longer processing times. Therefore, our focus was on the preparation and investigation of Ti_3_AlC_2_/Ni–Al, Ti_3_AlC_2_/Ni–Cu–Al and Ti_3_AlC_2_/Cu–Al composites (Fig. 2A–C and Table 1).

#### 3.1.1 Ti_3_AlC_2_/Ni–Al composites

An important factor influencing the final phase composition is the initial component ratio. Since several reactions can take place during the synthesis, the MAX phases typically coexist with carbides (TiC) and some intermetallic compounds (TiAl_3_). For Ti_3_AlC_2_ synthesis, a small excess of Ti and Al in the reaction mixture is typically used (Ti : Al : C = 2 : 1.1 : 1) to reduce TiC content in final products.^57^ The M–A bonds in the MAX phases and in Ti_3_AlC_2_, in particular, have a weak covalent character,^58^ with the Al 2p binding energy of 72.1 eV; for pure Al this value is equal to 72.8 eV.^59^ Ni forms several phases with Al, exhibiting different binding energies, including NiAl_3_, Ni_2_Al_3_, NiAl, Ni_3_Al. The binding energy of Al 2p level in NiAl_3_ alloy is 72.8 eV, which is identical to that of pure elemental Al. In the case of NiAl and Ni_3_Al alloys, it has been reported as 72.7 and 72.6 eV, respectively.^60^ It was shown that Ti_3_AlC_2_ and Ni_2_Al_3_, NiAl_3_ phases can coexist in one sintered composite, but the Al content must ensure the formation of both the MAX phase and NiAl_3_ or Ni_2_Al_3_ phases.^38,39^ Thus, a minimum of one mole of Al is required to obtain one mole of Ti_3_AlC_2_ MAX phase, and preferably three moles of Al to form the NiAl_3_ or Ni_2_Al_3_ phase to avoid TiC formation. At a certain Al/Ni ratio in the initial reaction mixture, the formation of Ti_3_AlC_2_ MAX phase and NiAl_3_, Ni_2_Al_3_ phases occurs, while at lower Al/Ni ratio the sintered products contain Ti_3_AlC_2_ MAX phase along with Ni_2_Al_3_, NiAl phases and TiC.^38^

The thermal effect of self-propagating high-temperature synthesis (SHS) allows to obtain Ti_3_AlC_2_ from initial elements Ti, Al, C, but sintered products also contain TiC and TiAl_3_.^61^ At high Al content in the reaction mixture (without the addition of Ni–Al alloys), even after heating at 1200 °C for 1 hour a considerable content of TiC and TiAl_3_ in reaction products is observed (sample 1, Fig. 2A). The formation of Ni–Al compounds from elemental Ni, Al is an exothermic process which can proceed in the SHS mode.^62^ Therefore, the addition of Ni and Al facilitates Ti_3_AlC_2_ formation even when heating up to 800°,^38^ while high Al content in the initial reaction mixture leads to higher TiAl_3_ and TiC content in final products (samples S1, S2, Fig. S1A).

It was shown that it is possible to reduce the amount of TiAl_3_ and TiC, decrease sintering temperature and increase the uniformity of Ti_3_AlC_2_ crystals by increasing the sintering time at a lower temperature. In XRD data of samples sintered at 1200 °C for 1 hour, the peaks related to TiC were absent (samples 2, 3, Fig. 2A) which indicates that the presence of liquid phases (NiAl_3_, Ni_2_Al_3_) during sintering promotes Ti_3_AlC_2_ MAX phase formation, allowing a reduction of the synthesis temperature compared to other synthesis methods (1300–1450 °C).^57,63,64^ Synthesis at a higher temperature (1450 °C) for 1 hour also leads to higher TiAl_3_ and TiC content in sintered products, which indicates that high temperature is not favourable for the formation of the Ti_3_AlC_2_ MAX phase.

#### 3.1.2 Ti_3_AlC_2_/Ni–Cu–Al composites

The simultaneous addition equal amounts of Ni and Cu to the reaction mixture with high Al content leads to the formation Ti_3_AlC_2_/(NiCu)_2_Al_3_ composites over a relatively wide range of component ratios (sample 4, 6, Fig. 2B). The liquid phase sintering effect for Ti_3_AlC_2_ MAX phase is also observed, however, at a higher (NiCu)_2_Al_3_ content the reaction products contain more TiC (sample 6). Similar to Ti_3_AlC_2_/Ni–Al composites, the optimal synthesis temperature was chosen at 1200 °C, since a more complete conversion of the initial components into the MAX phase occurred (samples S3, S4, S5, Fig. S1B).

#### 3.1.3 Ti_3_AlC_2_/Cu–Al composites

In order to obtain Ti_3_AlC_2_/Cu–Al composites, different ratios of the components Cu : Ti : Al : C were tested. Various crystalline phases may form in the Cu_1−*x*_Al_*x*_ system.^65^ Cu–Al alloys with high Al content have a relatively low melting temperature (550 °C for the CuAl_2_ phase), and can play the role of solvent for other components in the reaction mixture. Therefore, the presence of Cu–Al phases has an impact on Ti_3_AlC_2_ phase formation during sintering (Fig. 2C).

The binding energy of Al in CuAl_2_ is slightly stronger (72.93 eV for Al 2p_3/2_ and 73.33 eV for Al 2p_1/2_)^66^ than in Ti_3_AlC_2_, although these values were taken at room temperature. Therefore, the amount of Al in the initial reaction mixture should be sufficient to form Ti_3_AlC_2_ and CuAl_2_ phases while avoiding the formation of TiC. CuAl_2_ melt facilitates Ti_3_AlC_2_ MAX phase crystal growth and promotes its formation at lower temperature. Thus, Ti_3_AlC_2_/Cu–Al composites can be obtained even by sintering at 1000 °C for 4 hours (sample S6, Fig. S1C).

It is known that Cu–Al dealloying in HCl or NaOH allows the formation of hierarchical nanoporous copper structures.^67^ After etching of bulk Ti_3_AlC_2_/Cu–Al composites in HCl solution (15 wt% aqueous solution), peaks related to Ti_3_AlC_2_ and metallic copper are observed in XRD data of the samples 7a, 9a (Fig. 2D). Peaks related to TiC in XRD data are absent, indicating high conversion of TiC to Ti_3_AlC_2_ MAX phase.

Therefore, in general, Ti_3_AlC_2_ MAX phase synthesis in the presence of metal–Al alloys presents notable advantages. Cu–Al, Ni–Al and Ni–Cu–Al alloys with high Al content have lower melting temperatures than TiAl_3_ (Ni_2_Al_3_*T*_melt_ = 1133 °C, NiAl_3_*T*_melt_ = 854 °C,^62^ CuAl_2_*T*_melt_ = 550 °C,^68^ TiAl_3_*T*_melt_ = 1340 °C).^69^ The presence of these phases in the reaction mixture accelerates Ti_3_AlC_2_ MAX phase formation process and reduces the synthesis temperature and time. Compared to the molten salt method for MAX phases synthesis, where reactions and component diffusion are facilitated by a molten salt,^70^ synthesis in molten metal–Al alloys offers several advantages. The higher solubility and diffusion rates of the components such as TiAl_3_ and Al_4_C_3_ allow for a broader sintering temperature range, as it is not constrained by the decomposition temperature of salts.^71^ This is particularly important for the synthesis of high-melting-point MAX phases, for example, containing Mo and Nb.

#### 3.1.4 Structural characterisation

The indexed XRD diffraction patterns of samples 7a and 9a (samples 7 and 9 after etching in HCl solution) are shown in Fig. 2D (linear scale) and Fig. 2E (logarithmic scale). The summary of the XRD results is presented in Table 2 (the normalized peak intensities, relative to the 002 peak as the most intense). To the authors’ knowledge, this is the first quantitative presentation of the XRD results for Ti_3_AlC_2_ MAX phase obtained in the presence of metal–Al (Cu–Al) alloys. The XRD results are shown in comparison with those previously reported by N. V. Tzenov^63^ and M. A. Pietzka^72^ for individual phases.

**Table 2:** Summary of XRD data obtained for MAX phase/Cu composites

*hkl*	Measured 2*θ* value (°)		
	M. A. Pietzka^63^	N. V. Tzenov^72^	This work
002	9.53	9.525	**9.50**
004	19.09	19.17	19.03
006	28.81		28.70
100	33.63	33.74	
101	33.98	34.07	33.84
102	35.00	35.16	34.88
103	36.70	36.82	36.55
008	38.74	38.85	**38.40**
104	38.96	39.07	**38.64**
105	41.68	41.85	41.53
106	44.84	44.96	44.67
107	48.38	48.59	48.19
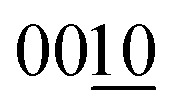	48.99		
108	52.22	52.46	52,03
109	56.34	56.62	56.13
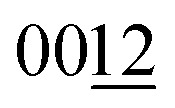	59.68		
110	60.13	60.31	**59.87**
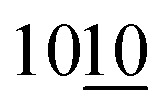	60.34		
112	61.02	61.06	60.51
114	63.70		
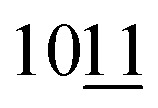	65.46	65.73	65.13
116	68.00		
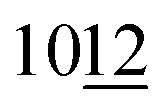	70.27	70.63	70.00

The diffraction patterns of such composites contain peaks related to the Ti_3_AlC_2_ MAX phase, Cu, and impurities (Al_2_O_3_, AlCu_2_Ti). There are several differences between the presented diffraction patterns and the literature data. A noticeable shift of most diffraction peaks belonging to the Ti_3_AlC_2_ toward smaller diffraction angles is observed, while the position of the diffraction peaks characteristic of Cu, Al_2_O_3_ and AlCu_2_Ti has not changed. A similar shift of the diffraction peaks is also observed for the samples sintered with Ni–Al and Ni–Cu–Al. The lattice parameters *a* = 0.3085 nm and *c* = 1.853 nm, calculated herein from the (110) and (002) peaks are slightly larger than those reported previously.^63,72^ This is likely due to the synthesis conditions, which occur under pressureless liquid-phase conditions (metal–Al alloy), reducing the mutual influence of the MAX phase crystals on each other.

The high intensity of several peaks (002), (004), (008) compared to other peaks related to MAX phase indicates that the samples are textured, *i.e.*, the crystals are preferentially oriented parallel to the *a*-axis.^73^ In the samples obtained after etching in HCl most of the MAX phase crystals are separated from each other, leading to crystals ordering and formation of textured samples during the deposition and alignment of the powder onto the substrate for recording the diffraction pattern.

In the obtained samples, the intensities of the (002) peak at 2*θ* = 9.5° and the (004) peak at 2*θ* = 19° are higher than the peaks attributed to reflection from other planes, compared to literature data.^63,72^ The increase in the intensity of these peaks is a result of the reflection of X-ray radiation from a set of crystallographic planes, which is due to an increase in the number of layers in an individual crystallite of the MAX phase.^74^

It should be noted that the diffraction pattern in the region of small angles also contains two new peaks (marked with “*x*”) at 2*θ* = 8.57° and 9.09° (Fig. 2E) and are most likely attributed to the partial formation of MXene as a result of Al etching in HCl solution along the perimeter of the MAX phase crystals. Also, these new peaks may also correspond to new phases.

### 3.2 Structural and microstructural characterisation of MXene/metal composites

#### 3.2.1 MXene/(NiCu)_2_Al_3_ composites

After etching of Ti_3_AlC_2_/Ni–Al composites (samples 2, 3) in HF solution, Ni and Al in Ni–Al alloys undergo dissolution, leading to the formation of MXene with high degree of ordering. However, high content of corrosion-resistant (NiCu)_2_Al_3_ phase in Ti_3_AlC_2_/(NiCu)_2_Al_3_ composites inhibits Al etching in HF solution (sample 6). Thus, at low (NiCu)_2_Al_3_ phase content after etching in HF solution, this phase remains in the etching products, forming MXene/(NiCu)_2_Al_3_ composites (sample 4b and 4c, Fig. 2F). It should be noted that (NiCu)_2_Al_3_ phase is stable in solution of HF, but in HCl solution Ni and Al from (NiCu)_2_Al_3_ phase also undergo dissolution. In alkaline conditions, (NiCu)_2_Al_3_ can be decomposed with the formation of nanoporous Ni–Cu compounds and Al_2_O_3_ (sample 4c, Fig. 2F).

**Fig. 3:**
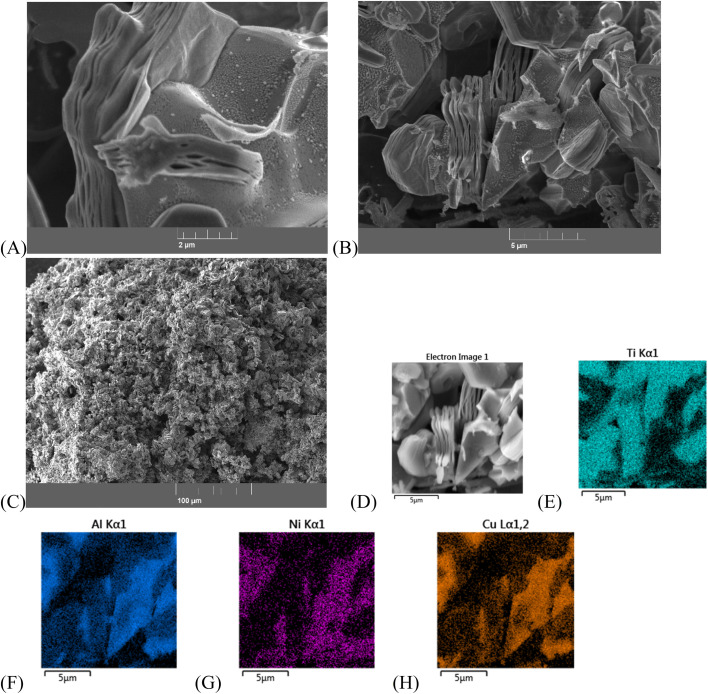
SEM images of the MXene/(NiCu)_2_Al_3_ composite (sample 4b): SEM images (A–C), SEM/EDS mapping of the interface showing the distribution of Ti (light blue colour), Al (blue colour), Ni (lilac colour), Cu (orange colour) in the surface layer (D–H).

SEM studies of sample 4 after etching in HF solution show MXene crystals combined with (NiCu)_2_Al_3_ alloy (sample 4b, Fig. 3A–C). An intercrystalline porous structure is formed by the phases that undergo etching in HF solutions. These phases are present in initial sintered products, but are dissolved by etching (TiAl_3_, Al). SEM/EDS mapping allows identification of the distribution of elements in composite structure. As expected, Ti is mainly present in MXene and Ni, Cu, Al in (NiCu)_2_Al_3_ alloy (Fig. 3D–H).

#### 3.2.2 MXene/Cu composites

After etching of Ti_3_AlC_2_/Cu–Al composites with low Cu–Al alloys content (sample 7) in HCl solution, porous Ti_3_AlC_2_/Cu composites are formed. A hierarchical 3D nanoporous copper that forms during etching of samples 8, 9, 10 with high Cu–Al alloys content facilitates Al etching and allows the formation of bulk MAX phase Ti_3_AlC_2_/Cu porous structure without milling and destruction of sintered samples (SEM images of sample 10a, Fig. S2A and B).

Further etching of this Ti_3_AlC_2_/Cu samples in HF solution allows to obtain MXene/Cu materials with a high degree of ordering sample 10b (Fig. 2F) and samples 7b, 9b (Fig. S1D). An intercrystalline porous structure is formed not only after etching of the TiAl_3_ and Al, but also of the Cu–Al, Al–Cu–Ti phases, similar to described in the literature for the porous copper structure obtained by dealloying of Al–Cu–Ti compounds.^75^

Al etching of Ti_3_AlC_2_/Cu–Al composites under alkaline conditions (10 M NaOH at 70 °C for 10 days) also results in the formation of composite nanoporous MAX phase/MXene/Cu materials with a high intercrystallite porosity. However, in this case the MXene content was relatively low due to incomplete Al removal from the Ti_3_AlC_2_ MAX phase.

In fact, several types of MXene/Cu composites presented in the literature can be distinguished. These include Cu in the form of ∼5 nm nanoparticles,^76^ single Cu atom doped into MXene flakes deposited from CuCl_2_ salt solution^8,77^ or obtained *via* molten salts MAX phase etching.^78^ All factors such as copper concentration, deposition potential, solution pH and the presence of a background electrolyte have profound effects on the nucleation mechanisms.^79^

**Fig. 4:**
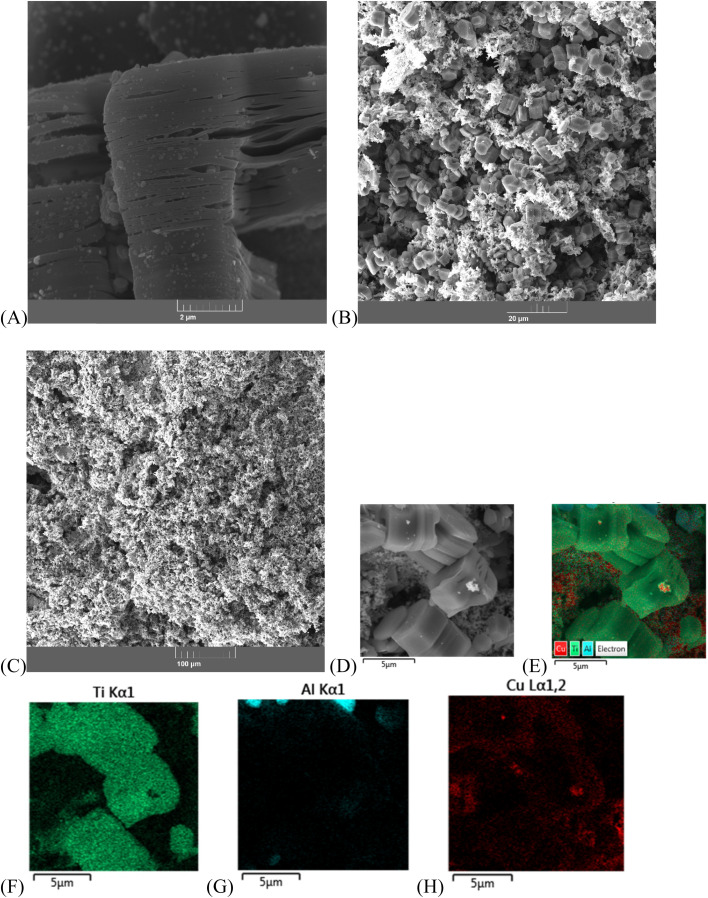
SEM images of the MXene/Cu composite: sample 10b (A–C), SEM/EDS mapping of the interface of sample 10b showing the distribution of Ti (green colour), Al (blue colour), Cu (orange colour) in the surface layer (D–H).

SEM studies of MXene/Cu samples after etching showed that, at low Cu content after Al etching, Cu is mainly present in the form of nanoparticles on the surface of MXene crystals. At higher Cu content, such composites exhibit good mechanical strength and form a monolithic three-dimensional porous structure consisting of MXene crystals and nanoporous metallic Cu (sample 10b, Fig. 4A–C). SEM/EDS mapping allows identification of the distribution of elements in composite structure, Ti is present in MXene, and Cu on the surface and between MXene crystals (Fig. 4D–H).

Cu is a relatively unreactive metal, but in oxidizing environments at low pH it may dissolve forming Cu^+^ ions.^80^ Compared to bulk copper, porous Cu reacts with HF solution and acts as a source of Cu ions. In turn, Ti-vacancies, defects, and –OH groups can act as active sites for Cu ions adsorption.^8^ Therefore, single Cu atom dopants can also be present in obtained samples. Cu deposition on MXene and formation nanoparticles takes place by an electrochemical reduction mechanism analogous to Cu ions reduction on Si surface.^81^

The work function of Ti_3_C_2_ MXene varies over a wide range, depending on the surface functional groups and composition. The typical value for Ti_3_C_2_(OH, F)_*x*_ MXene is approximately 3.9 eV,^82^ making it suitable for Cu ion reduction. The MXene may act as a reductant during the reaction process, causing the formation of Ti^3+^.^83^ F Further Cu-ion deposition occurs predominantly on existing nuclei, because Cu-ion reduction at the MXene/HF interface occurs much more slowly than at the metal/HF junction. In MXene/Cu samples the deposited Cu nanoparticles possess a broad size distribution. The presence of big nanoparticles (up to 200 nm) likely due to prolonged Cu deposition process (two-stage Ti_3_AlC_2_/Cu etching in HF solution, for 48 hours). An important issue is that Al etching and Cu deposition occurred simultaneously in a single step. This process allows the use of Al as a reducing agent (sacrificial electrode), preventing MXene oxidation and Ti^3+^ formation. EDX elemental analysis reveals the presence of Ti, C, O, F, and Cu in the obtained MXene/Cu composites (Fig. S3).

Etching of bulk sample 5 (Ti_3_AlC_2_/(NiCu)_2_Al_3_) in HF solution or bulk samples 8, 9, 10 (Ti_3_AlC_2_/Cu–Al) in HCl and then in HF solution, results in the formation of porous layer of MXene/(NiCu)_2_Al_3_ or MXene/Cu composites on the surface. In such cases, nanoporous Cu or (NiCu)_2_Al_3_ phase act as a support or binding material for MXene crystals.

### 3.3 Electrochemical characterisation

The specific electrochemical properties (electrocatalytic activity, capacitance) of MXene-based electrodes are highly dependent on several factors, including the ionic diffusion resistance and electrical conductivity of active material among the main factors. These, in turn, depend on the porosity and thickness of the active material film. The specific capacitance drops three times with an increase in film thickness from 5 to 75 μm in thick electrodes.^84^ Therefore, in the literature, the electrochemical tests of the MXene-based materials are usually conducted with a relatively low mass loading per unit area of the coating, amounting to 5–10 mg cm^−2^ and thickness of several micrometres.^85^ For thick films (∼100 μm) of active material, different additives are used to create a conductive network between the MXene flakes, such as carbon^86^ and noble metal nanoparticles (1–20 nm).^28,83,87^

#### 3.3.1 MXene/metal composites (powders)

In this work, comparative tests were carried out for MXene and MXene/metal composites (in the form of compacted powders), with a mass density per unit area of approximately 37 mg cm^−2^. Taking into account the average pore size of ∼200 μm for the copper foam substrate, this value can be roughly compared to a virtual thickness of an active-material film of ∼100 μm.

**Fig. 5:**
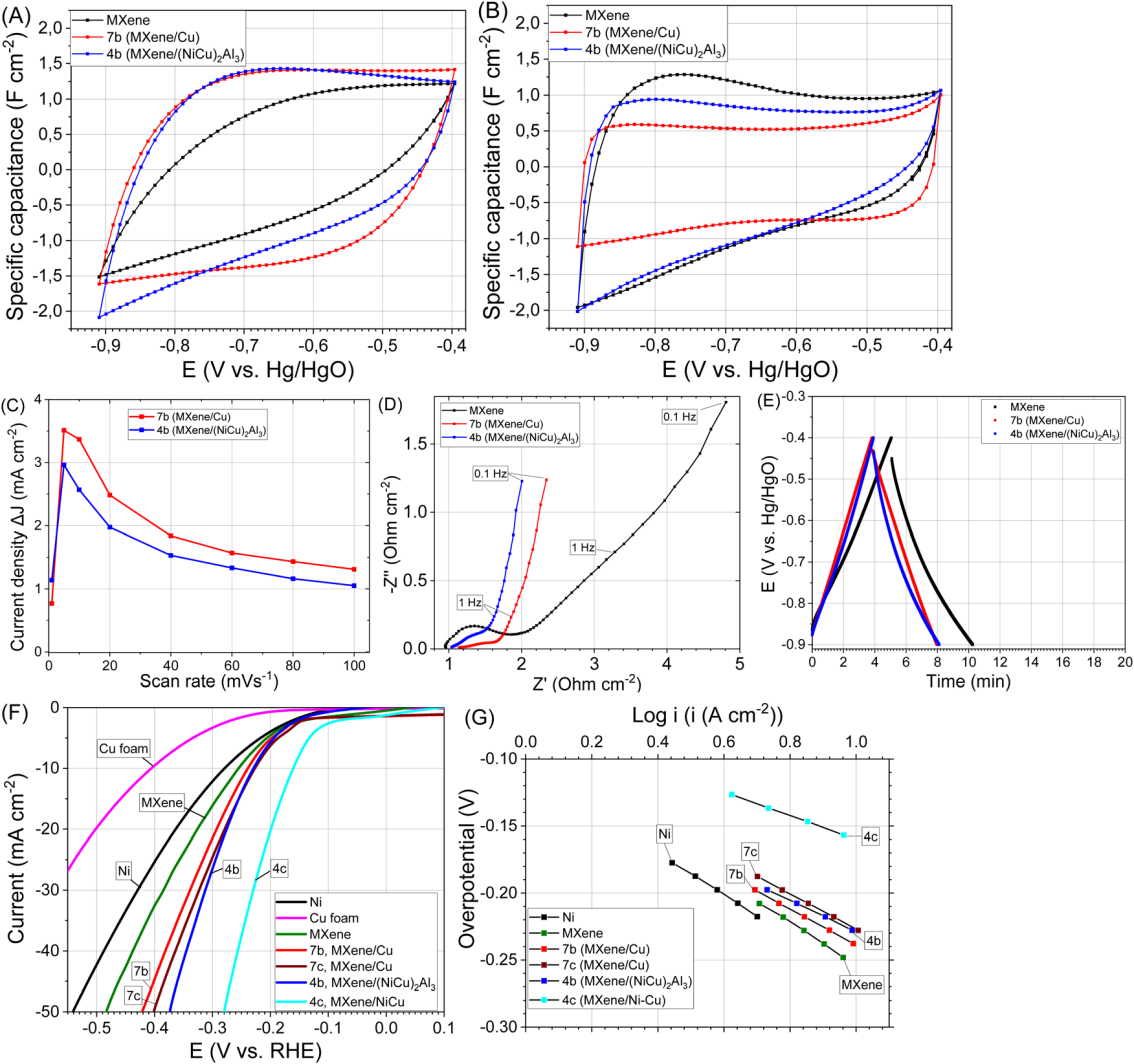
Electrochemical performance of samples 7b (MXene/Cu), 4b (MXene/(NiCu)_2_Al_3_) and bare MXene: CV curves of electrodes at scan rate 10 mV s^−1^ (A), CV curves of electrodes at scan rate 1 mV s^−1^ (B), the differences in current density plots against scan rate (C), electrochemical impedance spectroscopy data (D), galvanostatic cycling data collected at 0.08 A g^−1^ (E), HER polarization curves recorded at scan rate 1 mV s^−1^ (F), Tafel plots for the HER occurring on the electrodes (G).

The samples of MXene/Cu, MXene/(NiCu)_2_Al_3_ composites demonstrate higher capacitance compared to bare MXene at a scan rate 10 mV s^−1^ and slightly lower at 1 mV s^−1^. This behaviour is caused by the improved electrical conductivity of active material in MXene/metal electrodes (Fig. 5A and B). In CV curves, the profiles are non-rectangular and display humps or redox peaks, especially for MXene/Cu and MXene/(NiCu)_2_Al_3_ composites. This indicates that faradaic (pseudocapacitive or redox) reactions contribute significantly to the total current. For non-ideal CVs, especially with sloped or peaked profiles, the integration no longer isolates capacitive charge from faradaic charge properly. To accurately quantify the capacitance when the CV is non-rectangular, we used an average current method at a fixed potential region where the behavior is mostly capacitive (at −0.75 V). Cu in alkaline media can undergo electrochemical oxidation and reduction, forming different products depending on the applied potential.^88,89^ The formation of Cu(OH)_2_^−^, Cu_2_O compounds can occur when the potential is scanned from negative to positive in the range from −0.5 to −0.4 V (*vs.* Hg/HgO), the corresponding oxidation peaks are observed.^88^ Two peaks also appear in the reverse scan, corresponding to the reduction of the oxidized species (Cu from Cu_2_O and Cu(OH)_2_^−^). As can be seen in CV curves of obtained composites, such oxidation/reduction peaks are not visible at 10 mV s^−1^ (Fig. 5A) and the influence of redox processes becomes noticeable in the range from −0.6 to −0.4 V at a low scan rate (1 mV s^−1^) (Fig. 5B). Cu_2_O is a solid substance; therefore, it can be assumed that it undergoes reduction at the same sites on the electrode during the reverse scan. Cu(OH)_2_^−^ ions are soluble, although the extent of the dissolution reaction is very small.^88^ During the reverse scan, the reduction and deposition of Cu occur over the entire electrode surface. The stability test of sample 7b was conducted for 10 days (∼4130 cycles charge/discharge, from −0.9 to −0.4 V *vs.* Hg/HgO, at scan rate 5 mV s^−1^) and only a 3% decrease in capacitance was observed.

The (NiCu)_2_Al_3_ phase in the MXene/(NiCu)_2_Al_3_ composite is less stable in 1 M NaOH solution and undergoes partial decomposition with the formation of porous Ni–Cu compounds and Al_2_O_3_. In addition, after CV cycling of sample 4b for 10 days, a layer of deposit on the negative electrode (Cu foam substrate) was observed indicating that Cu from the (NiCu)_2_Al_3_ phase undergoes dissolution followed by redeposition from the electrolyte. Ni in alkaline conditions is present in the form of metallic Ni and Ni(OH)_2_; during cycling the conversion of Ni(OH)_2_ species to metallic Ni occurs^39,90,91^ Ni and Cu in sample 4b are present mainly in metallic form and provide electronic conductivity. A 5% decrease in capacitance was observed likely resulting from the volume expansion of the Al_2_O_3_ phase between the MXene crystals, which creates diffusion limitations for the electrolyte ions.

The weight ratio of MXene in composites is lower (in sample 7b MXene ∼77 wt%, and Cu ∼23 wt%, in sample 4b MXene ∼68 wt%, and (NiCu)_2_Al_3_ ∼32 wt%). Therefore, the values of gravimetric capacitance for obtained composites at scan rate 10 mV s^−1^ are close (38 F g^−1^ for sample 7b, and 34 F g^−1^ for sample 4b), but slightly lower than those reported in the literature for MXene, where capacitance is mainly provided by Na^+^ ion intercalation mechanism in MXene (∼50 F g^−1^ at scan rate 10 mV s^−1^).^86^

ECSA calculations from the corresponding CV data (recorded at OCP −0.75 V *vs.* Hg/HgO, Fig. S4A, B) are complicated by a considerable increase in current density at low scan rate (Fig. 5C). This behaviour might be promoted by a large amount of nanopores formed by interlayer space of MXene. These pores become accessible for electrolyte ions (Na^+^) only at a low scan rate, resulting in an increase in the current density. The insertion of sodium ions enhances the overall capacitance, allowing for greater energy storage.^92^ The calculated values of ECSA are listed in Table 3. Values calculated at high scan rates (100 mV s^−1^) are likely related to the outer surface area of MXene crystals and the porous structure formed by the metallic phases (porous Cu, (NiCu)_2_Al_3_) in the samples, without intercalation of electrolyte ions (Na^+^) between the MXene layers. Values obtained at low scan rates (1–10 mV s^−1^) also include the contribution of the MXene interlayer space (electric double-layer capacitance combined with pseudocapacitance) and are comparable with literature values.^93^ The MXene/Cu composite demonstrates higher ECSA values than MXene/(NiCu)_2_Al_3_, which is attributed to the higher MXene content and the presence of nanopores in the metallic Cu framework of the MXene/Cu composite.

**Table 3:** Estimated values of capacitance (C1) from CV curves (at 10 mV s^−1^ from −0.9 to −0.4 V) and ECSA from CV curves for samples (at 10 mV s^−1^)

Samples	C1 (F cm^−2^)	C1 (F g^−1^)	ECSA (m^2^ cm^−2^)	ECSA (m^2^ g^−1^)
MXene	0.86	27		
4b, (MXene/(NiCu)_2_Al_3_)	1.27	34	0.64	4.94
7b, (MXene/Cu)	1.37	38	0.84	6.47
5b bulk, (MXene/(NiCu)_2_Al_3_)	0.65		0.26	
8b bulk, (MXene/Cu)	0.69		0.30	
10b bulk, (MXene/Cu)	1.7	9.8	0.43	2.3

To evaluate the electrical conductivity of samples 4b and 7b, impedance spectra were recorded. The Nyquist plots revealed lower electrical impedance for MXene/metal composites compared to bare MXene (Fig. 5D).

The GCD profile of the MXene/Cu composite (sample 7b) exhibited a nearly triangular shape, indicating reversible cation intercalation during the charge–discharge process (Fig. 5E).

#### 3.3.2 MXene/metal bulk composites

At low metal content in MXene/metal composites the porous MXene/metal layer on the Ti_3_AlC_2_/metal–Al surface (bulk samples 8b, 5b) exhibited poor mechanical strength. Therefore, only partially etched bulk samples (8b, 5b), with MXene/metal thickness of several tens of micrometres, were used for electrochemical testing. The weak mechanical strength of bulk samples 8b, 5b facilitates MXene delamination, however, when the etched layer thickness increases, the material tends to disintegrate.

**Fig. 6:**
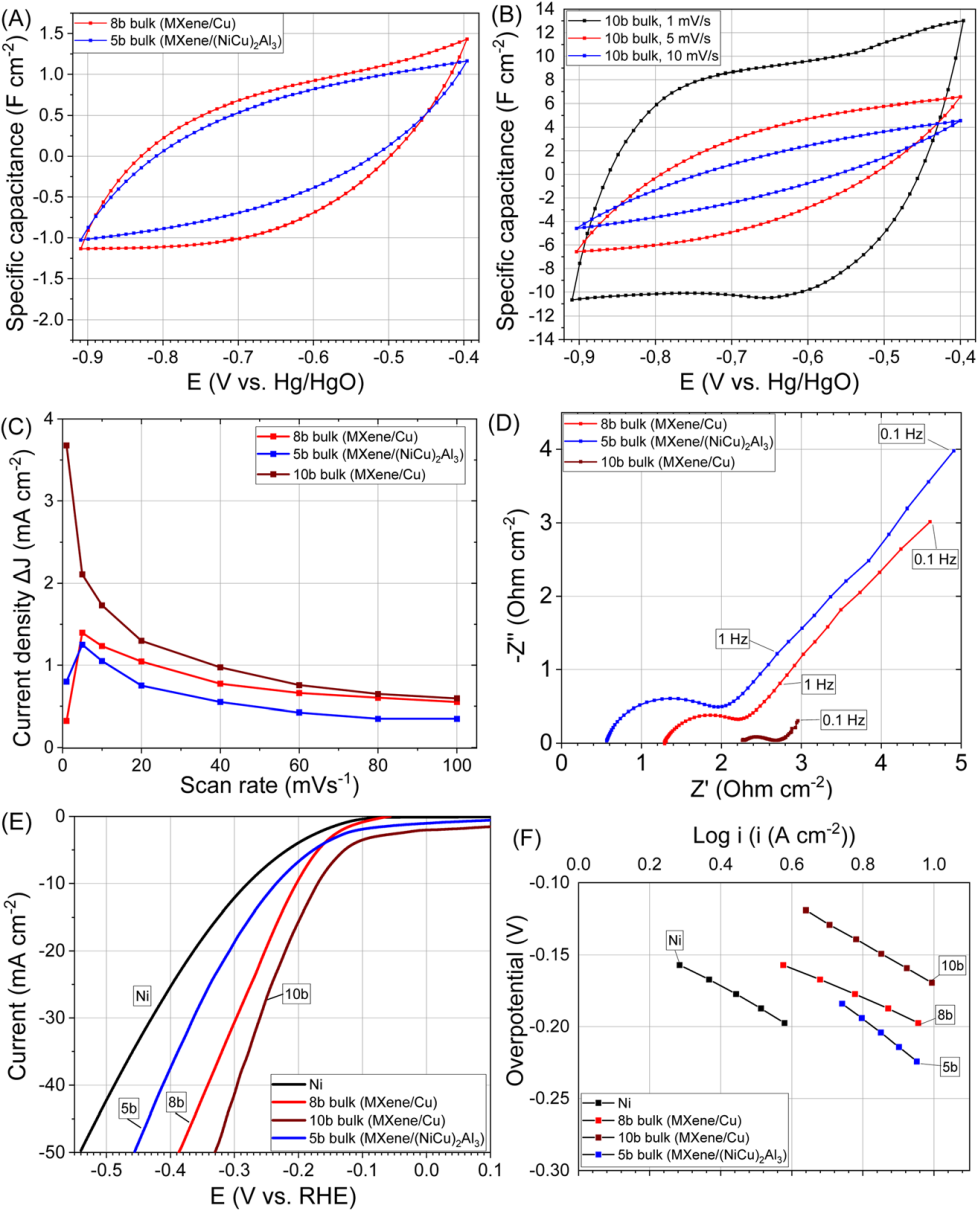
Electrochemical performance of bulk 8b, 10b (MXene/Cu) and 5b (MXene/(NiCu)_2_Al_3_) samples: CV curves of electrodes at scan rate 10 mV s^−1^ (A), CV curves of electrode 10b at scan rate 1, 5, 10 mV s^−1^ (B), the differences in current density plots against scan rate (C), electrochemical impedance spectroscopy data (D), HER polarization curves (E), Tafel plots for the HER occurring on the electrodes (F).

At higher Cu content fully etched MXene/Cu composite (bulk sample 10b, 4 mm thickness, 17 mm diameter) have sufficient mechanical strength and is a three-dimensional porous composite. Areal capacitance (Fig. 6A and B), the differences in current density plots against scan rate (Fig. 6C, and S5A–C), and conductivity (Fig. 6D) of bulk samples are largely determined by the thickness of MXene/metal layer (Table 3). At higher Cu content, the resulting MXene/Cu structure (bulk sample 10b) exhibited improved mechanical stability and, consequently, higher areal capacitance (Fig. 6B). The maximum capacitance value was observed at a low scan rate of 1 mV s^−1^, reaching 9 F cm^−2^ (Fig. 6B). The plots of current density *versus* scan rate also reached their maxima at low scan rates, which is attributed to the bulk porous Cu matrix restricting expansion between MXene layers even after TBAOH delamination. This limitation hinders deep electrolyte ion intercalation between individual MXene sheets. The mass of bulk sample 10b was 2.4 g, consisting of 1.53 g Cu (64 wt%) and 0.87 g MXene (36 wt%). Accordingly, the gravimetric capacitance of the composite was approximately 18 F g^−1^ at 1 mV s^−1^. A significant part of this capacitance is provided by MXene, although the CV curves also display peaks associated with oxidation–reduction processes of nanostructured Cu (Fig. 6B).

### 3.4 Hydrogen evolution tests and surface analysis

Bare (unmodified) MXenes, such as Ti_3_C_2_T_*x*_, generally exhibit only moderate HER activity, particularly when compared with optimized heterostructures.^85^ Hydrogen evolution tests revealed lower overpotential for the obtained MXene/metal composites compared to bare MXene, Ni or Cu electrodes (Fig. 5G and H). Determination of the HER reaction mechanism and the rate-determining step (RDS) can be performed using Tafel analysis, although the derived parameters should be considered approximate because the Tafel slope depends on the coverage of the surface intermediates.^94^ For this purpose, the polarization curves were linearized according to the Tafel equation:

1 *η* = *a* + *b* log(*j*)

where *η* is the overpotential (V), *j* is the current density (A cm^−2^), *a* and *b* are the Tafel constants. The Tafel slope (*b*) provides information about the electrochemical mechanism, while a reflects the intrinsic electrocatalytic activity of the electrode surface. Typically, Tafel slopes of 120, 40, and 30 mV dec^−1^ correspond to the Volmer, Heyrovsky, and Tafel rate-determining steps, respectively. For pure Ni, the Volmer step (water dissociation) is often the slowest, leading to Tafel slopes in the range of 90–120 mV dec^−1^. Current densities between 1 and 10 mA cm^−2^ are commonly employed, as this range lies within the activation-controlled region and avoids high overpotentials where ohmic or mass-transport effects become significant. The kinetic parameters of HER, calculated from the Tafel curves, are summarized in Table 4. According to the Tafel analysis, the Volmer reaction is the RDS for all investigated samples. However, accurate calculation of Tafel parameters (Tafel slope and exchange current density) in MXene-containing samples is complicated by the capacitive current contribution; therefore, these parameters were determined at a low scan rate (0.1 mV s^−1^) to minimize non-faradaic effects.

**Table 4:** Kinetic parameters of the HER for samples, calculated from voltammograms at 1–10 mA cm^−2^, recorded in 1 M NaOH solution

Samples (electrodes)	Tafel slope, *b* (mV dec^−1^)	The exchange current density, *j*_0_ (mA cm^−2^)	Transfer coefficient, *α*	Overpotential, *V*	
				10 mA cm^−2^	50 mA cm^−2^
Ni	156	0.020	0.37	−0.28	−0.54
MXene	159	0.025	0.36	−0.25	−0.48
7b, (MXene/Cu)	133	0.016	0.43	−0.23	−0.42
7c, (MXene/Cu)	130	0.016	0.44	−0.23	−0.40
4b, (MXene/(NiCu)_2_Al_3_)	117	0.013	0.49	−0.23	−0.37
4c, (MXene/(Ni–Cu))	88	0.090	0.66	−0.16	−0.28
5b bulk, (MXene/(NiCu)_2_Al_3_)	191	0.009	0.31	−0.23	−0.45
8b bulk, (MXene/Cu)	106	0.012	0.55	−0.20	−0.38
10b bulk, (MXene/Cu)	161	0.110	0.56	−0.17	−0.33

MXene/Cu composite (sample 7b) exhibited enhanced catalytic activity toward HER. Copper alone is not an efficient HER catalyst because of its weak hydrogen binding energy (HBE) (Fig. 5G and H). Therefore, the improved activity originates from the synergistic combination of several effects. In nanoporous structures, defect sites, surface strain, and undercoordinated Cu atoms modify the d-band structure, thereby enhancing HER activity. Also improved electrical conductivity between MXene layers in samples 4b, 7b appears to be a crucial factor. Electronic interactions at the MXene/metal interface can alter the electronic structure and catalytic behaviour of the metal (Cu). Delocalization of electrons within the MXene layer promotes electron transfer across the interface, modifying the metal's surface electronic states.^95^ In MXene/Cu composites, electron density is shifted from Cu to Ti_3_C_2_ which leads to the formation of electron-deficient Cu nanoparticles on the MXene surface, increasing the adsorption of active hydrogen atoms and enhancing HER performance.^76^ This interfacial charge redistribution is therefore identified as another major factor responsible for the high catalytic activity observed in sample 7b.

In the initial sample 10, which contained a high amount of copper, Al–Cu–Ti phases were observed (Fig. 2C), these phases may also be present in sample 7 with a lower copper content. Dealloying of Al–Cu–Ti compounds leads to the formation of Cu-based materials with two type pores: micropores that remain after removal of the Al-rich region and nanopores after removal of Al atoms from CuAl_2_ and Al–Cu–Ti compounds.^75^ This hierarchical porosity contributes to the high hydrogen-evolution activity, as it provides a large surface area for electrocatalytic hydrogen evolution and improves mass-transport properties. Nanoporous Cu often contains Cu_*x*_O species, which can facilitate water dissociation, thereby improving HER kinetics.

The HER stability test for sample 7b was conducted over 20 days (at −1.1 V *vs.* Hg/HgO, current density ≈10 mA cm^−2^). A slight increase in catalytic activity was observed (sample 7c), which was likely due to partial Cu dissolution (formation of Cu(OH)_2_^−^ ions in an oxygen environment) and subsequent redeposition of Cu nanoparticles on the MXene surface. This process likely resulted in a more uniform distribution of Cu over the MXene.

The MXene/(NiCu)_2_Al_3_ composite (sample 4b) shows slightly lower initial catalytic activity than the MXene/Cu composite (sample 7b), but higher than pure MXene. This difference can be attributed to the lower MXene content (∼68 wt%) and the presence of Ni and Cu in the corrosion-resistant (NiCu)_2_Al_3_ phase, which exhibits low porosity in the initial sample 4b.

After the 20 day stability test (at −1.1 *vs.* Hg/HgO) the catalytic activity of sample 4b increased gradually (sample 4c). XRD analysis of sample 4c (Fig. 2F) revealed that the (NiCu)_2_Al_3_ phase underwent partial decomposition under alkaline conditions, forming Al_2_O_3_ and Ni–Cu phases. Compared with literature data where Ni–Cu compounds are typically obtained by the reduction of Cu^2+^ and Ni^2+^ ions from solution the Ni–Cu phases produced *via* direct etching of (NiCu)_2_Al_3_ in 1 M NaOH should exhibit greater homogeneity. This is due to the mismatch in reduction potentials between Cu^2+^ and Ni^2+^ ions in solution and the faster deposition rate of Cu^2+^ relative to Ni^2+^.^5,96^ Taking into account the (NiCu)_2_Al_3_ phase structure,^97^ Al_2_O_3_ forms around the Ni and Cu atoms, acting as a substrate and providing stabilization of the nanostructured Ni–Cu phase against aggregation.

**Fig. 7:**
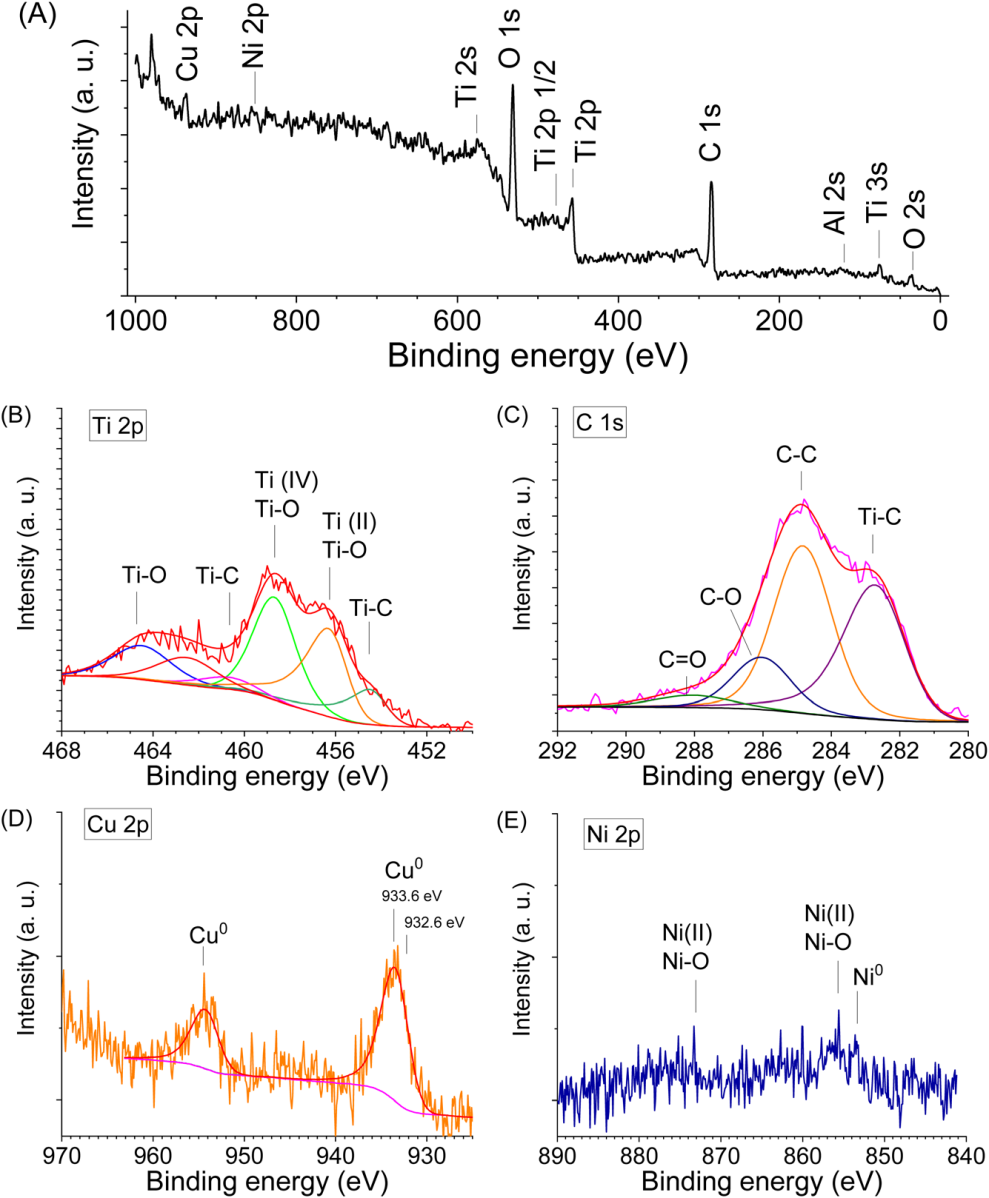
XPS spectra for the sample 4c (MXene/(NiCu)_2_Al_3_/Ni–Cu) after HER tests: the survey spectrum (A), the Ti 2p signal (B), the C 1s core level spectrum (C), the Cu 2p core-level spectrum (D), the Ni 2p spectrum (E).

To obtain more information about sample 4c, XPS analysis was performed. The survey revealed clear signals from Ti, C, O, Ni, Cu, and Al confirming the multicomponent nature of the composite. The general XPS spectrum in the F 1s region showed the absence of F-terminated Ti species after etching in 1 M NaOH, indicating successful removal of surface fluorine groups (Fig. 7A).

The Ti 2p spectrum displayed a main Ti 2p_3/2_ peak at 454.7 eV, characteristic of Ti–C bonding and confirming the presence of Ti_3_C_2_ MXene (Fig. 7B). In addition, the spectrum exhibited several higher binding energy components corresponding to oxidized Ti species: Ti(ii) (Ti 2p_3/2_ 456 eV), Ti(iii) (Ti 2p_3/2_ 457 eV) and Ti(iv) (Ti 2p_3/2_ 458.6 eV). Which indicates partial oxidation of Ti_3_C_2_ MXene during electrolysis. These features indicate partial oxidation of the Ti_3_C_2_ MXene surface during electrolysis.^76^

The C 1s core level spectrum (Fig. 7C) showed a dominant peak at 282.6 eV, assigned to carbon in Ti_3_C_2_ carbide. The peak at ∼284.6 eV in the C 1s XPS spectrum of Ti_3_C_2_ MXene is typically attributed to adventitious carbon or graphitic C–C bonds, which may originate from surface contamination or residual carbonaceous species, rather than from the Ti_3_C_2_ structure itself. Additional peaks were detected at 286.0 eV, corresponding to C–O bonds, and at 288.0 eV, corresponding to C=O bonds.

The Cu 2p core-level spectrum (Fig. 7D) revealed a Cu 2p_3/2_ binding energy of 933.6 eV, which is +1.0 eV higher than that of metallic Cu (932.6 eV)^98^ and +0.6 eV higher than values typically reported for Cu nanoparticle/MXene composites (932.9 eV).^76^ This positive shift suggests electron transfer from Cu to Ti_3_C_2_ upon contact, consistent with strong interfacial electronic coupling between Cu and MXene. The increased binding energy of Cu 2p_3/2_ (933.6 eV) therefore reflects intimate Cu/Ti_3_C_2_ interactions and may be associated with a more homogeneous distribution of Cu species including single atoms and nanoparticles on the MXene surface, promoted by prolonged cycling during the stability test. Notably, no additional peaks corresponding to Cu oxides were detected, indicating that copper remains predominantly in the metallic state after electrochemical operation. Furthermore, in the Ni–Cu alloy, Cu atoms share electrons with the more electronegative Ni atoms, leading to partial electron withdrawal from Cu. This effect causes tighter binding of the Cu core electrons and, consequently, an increase in the observed Cu 2p_3/2_ binding energy.^99^

A weak Ni 2p signal was observed in the spectrum of sample 4c (Fig. 7E), with the main Ni 2p_3/2_ peak located at 852.6 eV, characteristic of metallic Ni. A secondary peak at 855.5 eV corresponds to Ni 2p_3/2_ of Ni^2+^ in Ni(OH)_2_ species.^100^ In alloy systems, Ni may exhibit minor shifts in binding energy, although these are typically small unless oxidation occurs. Overall, the chemical environments inferred from the XPS analysis are consistent with the structural features determined by XRD.

The relatively weak Ni 2p signal intensity compared with the Cu 2p signal in sample 4c indicates a higher surface concentration of Cu than Ni. According to XRD data, Ni and Cu in sample 4c are predominantly present as the (NiCu)_2_Al_3_ phase (Fig. 2F). It is likely that partial dissolution of surface Ni from the (NiCu)_2_Al_3_ phase occurred during HF acid etching. The remaining surface layer of this phase appears to be coated with metallic Cu and Ni–Cu alloy, which protects the underlying Ni and Al from further etching and contributes to the enhanced corrosion resistance in HF medium.

When MXene is partially oxidized, its surface terminations can be replaced or complemented by TiO_2_-like domains. Although XRD did not reveal distinct peaks corresponding to titanium oxides in sample 4c (Fig. 2F), the electronic conductivity of pristine MXene is expected to decrease upon oxidation. However, the incorporation of conductive metals such as Cu significantly enhances the electrical performance of the composite. The synergistic interaction between MXene sheets and Cu nanoparticles promotes the formation of efficient electron-transport pathways, maintaining high electrical conductivity even after partial MXene oxidation.

The calculated exchange current density values (*j*_0_) which represent the intrinsic catalytic activity for MXene and MXene/metal composites (samples 4b and 7b) showed comparable initial values at low overpotentials. However, at higher current densities (50 mA cm^−2^), the bare MXene electrode exhibited a larger overpotential, attributed to poor electrical connectivity between individual MXene nanosheets.

The exchange current density obtained for sample 4c is comparable to values reported for pure Ni–Cu compounds.^5^ Several factors contribute to the enhanced catalytic activity. Ni alone provides strong hydrogen adsorption, which can hinder desorption, whereas Cu weakens the hydrogen binding energy (HBE). The combination of Ni and Cu modifies the electronic structure of Ni, thereby optimizing hydrogen adsorption energy. The electronic interaction between Ni and Cu downshifts the d-band center of Ni, which enhances catalytic activity by promoting hydrogen desorption from Ni.^101,102^ In addition, the MXene component in MXene/metal composites facilitates water adsorption and dissociation, resulting in improved HER activity at higher potentials, consistent with acceleration of the Volmer step in the HER process.

#### 3.4.1 MXene/metal bulk composites

At low Cu content in MXene/Cu composites (bulk sample 8b), and (NiCu)_2_Al_3_ in MXene/(NiCu)_2_Al_3_ composites (bulk sample 5b) exhibit HER activity comparable to that of the powder-based samples 7b and 4b (Fig. 6E, F and Table 4). This behavior is largely determined by the thickness of the MXene/metal layer. However, these composites display poor mechanical stability and tend to fracture under HER testing at high current densities (100 mA cm^−2^).

At higher Cu content, the three-dimensional (3D) porous MXene/Cu composite (bulk sample 10b) demonstrates sufficient mechanical strength, maintaining structural integrity even at current densities up to 100 mA cm^−2^. The calculated exchange current density (*j*_0_) for this sample indicates high intrinsic catalytic activity and low overpotential at low current densities. However, as the current density increases, a gradual decrease in HER performance is observed (Fig. 6E, F and Table 4). This performance drop is attributed to mass-transport limitations arising from the densely stacked MXene layers, which restrict ion and gas diffusion, reduce electrolyte accessibility, and trap generated hydrogen bubbles within the porous structure, leading to an apparent loss in catalytic activity.

It is believed that there remains considerable potential for further optimization of the 3D microstructural architecture and MXene layer arrangement in such electrodes to achieve superior electrocatalytic performance in future designs.

## 4 Conclusions

This work presents a simple and efficient strategy for the synthesis of MXene/metal composites from MAX phase/metal–Al alloy precursors. The presence of Ni_2_Al_3_, NiAl_3_ and (NiCu)_2_Al_3_, CuAl_2_ phases with low melting points in the initial reaction mixture enabled a reduction in synthesis temperature and time for the formation of the Ti_3_AlC_2_ MAX phase. In addition, these phases suppressed Al evaporation during sintering and enhanced the density of the final products. Consequently, NiAl_3_ and CuAl_2_ alloys provided a favourable environment for the growth of highly ordered MAX phase crystals.

Supported MXene/metal composites with porous architectures were directly obtained through etching and dealloying of bulk MAX phase/metal–Al precursors, without the need for additional processing steps.

The resulting monolithic MXene/Cu composites with high Cu content exhibited a hierarchical 3D porous structure, ensuring efficient electrolyte accessibility, enhanced interflake electronic conductivity, and effective utilization of MXene intercalation capacitance. However, the rigid nanoporous Cu matrix limited interlayer expansion in MXene, which constrained ion and gas diffusion and slightly reduced catalytic activity at high current densities.

Electrochemical studies demonstrated that the MXene/Cu and MXene/Ni–Cu composite electrodes possess moderate HER activity, attributed to their hierarchical porosity, high surface area, and MXene-metal electronic interactions. The synergistic combination of nanoporous Cu and Ni–Cu phases with MXene significantly enhanced electrocatalytic performance and charge transport efficiency.

Overall, this study highlights the potential of MXene/metal composites as promising electrode materials for electrochemical energy conversion and storage applications. Future research should aim to optimize the 3D microstructure and tune interfacial interactions to further improve electrocatalytic activity, electronic conductivity, and long-term operational stability.

## Conflicts of interest

There are no conflicts to declare.

## Supplementary Material

RA-015-D5RA07113E-s001

## Data Availability

Data for this article are available at Zenodo repository at https://zenodo.org/records/15280748. Data supporting this article have been included as part of the supplementary information (SI). Supplementary information is available. See DOI: https://doi.org/10.1039/d5ra07113e.

## References

[cit1] Aneke M., Wang M. (2016). Appl. Energy.

[cit2] Luo Y., Chen G. F., Ding L., Chen X., Ding L. X., Wang H. (2019). Joule.

[cit3] Jones J. P., Prakash G. K. S., Olah G. A. (2014). Isr. J. Chem..

[cit4] Liu J., Peng W., Li Y., Zhang F., Fan X. (2020). Trans. Tianjin Univ..

[cit5] Gao M. Y., Yang C., Zhang Q. B., Yu Y. W., Hua Y. X., Li Y., Dong P. (2016). Electrochim. Acta.

[cit6] Mahmood N., Yao Y., Zhang J. W., Pan L., Zhang X., Zou J. J. (2018). Adv. Sci..

[cit7] Gouveia J. D., Morales-García Á., Viñes F., Illas F., Gomes J. R. B. (2020). Appl. Catal., B.

[cit8] Eid K., Lu Q., Abdel-Azeim S., Soliman A., Abdullah A. M., Abdelgwad A. M., Forbes R. P., Ozoemena K. I., Varma R. S., Shibl M. F. (2022). J. Mater. Chem. A.

[cit9] Gogotsi Y., Huang Q. (2021). Am. Chem. Soc..

[cit10] Zhu Q., Li J., Simon P., Xu B. (2021). Energy Storage Mater..

[cit11] Lipton J., Röhr J. A., Dang V., Goad A., Maleski K., Lavini F., Han M., Tsai E. H. R., Weng G. M., Kong J., Riedo E., Gogotsi Y., Taylor A. D. (2020). Matter.

[cit12] Kondarage Y. G., Naiduwawadu A., Wijesinghe I., Dharmasiri C. D. H., Firestein K. L., Liao T., Yan C. (2025). Composites, Part A.

[cit13] Zhang J., Kong N., Uzun S., Levitt A., Seyedin S., Lynch P. A., Qin S., Han M., Yang W., Liu J., Wang X., Gogotsi Y., Razal J. M., Zhang J., Seyedin S., Lynch P. A., Qin S., Wang X., Razal J. M., Kong N., Yang W., Uzun S., Levitt A., Han M., Gogotsi Y. A., Liu J. (2020). Adv. Mater..

[cit14] Jia L., Zhou S., Ahmed A., Yang Z., Liu S., Wang H., Li F., Zhang M., Zhang Y., Sun L. (2023). Chem. Eng. J..

[cit15] Lipatov A., Alhabeb M., Lukatskaya M. R., Boson A., Gogotsi Y., Sinitskii A., Lipatov A., Boson A., Sinitskii A., Alhabeb M., Lukatskaya M. R., Gogotsi Y., Gogotsi J Y. A. (2016). Adv. Electron. Mater..

[cit16] Xu S., Wei G., Li J., Ji Y., Klyui N., Izotov V., Han W. (2017). Chem. Eng. J..

[cit17] Wu Z., Shang T., Deng Y., Tao Y., Yang Q. H. (2020). Adv. Sci..

[cit18] Li P. X., Guan G. Z., Shi X., Lu L., Fan Y. C., Xu J., Shang Y. Y., Zhang Y. J., Wei J. Q., Guo F. M. (2023). Rare Met..

[cit19] Guo M., Liu C., Zhang Z., Zhou J., Tang Y., Luo S., Guo M., Liu C., Luo S., Zhang Z., Zhou J., Tang Y. (2018). Adv. Funct. Mater..

[cit20] Dahlqvist M., Barsoum M. W., Rosen J. (2024). Mater. Today.

[cit21] Abdisattar A., Yeleuov M., Daulbayev C., Askaruly K., Tolynbekov A., Taurbekov A., Prikhodko N. (2022). Electrochem. Commun..

[cit22] Zhang J., Li C. M. (2012). Chem. Soc. Rev..

[cit23] Xiong X., Ding D., Chen D., Waller G., Bu Y., Wang Z., Liu M. (2015). Nano Energy.

[cit24] Qiu H. J., Ito Y., Chen M. W. (2014). Scr. Mater..

[cit25] Komal N., Ali G., Sohail M., Mazhar M., Malik Z., Wattoo M. H. S. (2023). Mater. Chem. Phys..

[cit26] Fujita T. (2017). Sci. Technol. Adv. Mater..

[cit27] Banhart J. (2001). Prog. Mater. Sci..

[cit28] Zhang Q., Wang J. A., Yu Q., Li Q., Fan R., Li C., Fan Y., Zhao C., Cheng W., Ji P., Sheng J., Zhang C., Xie S., Henkelman G., Li H. (2024). Nat. Synth..

[cit29] Khan M. U., Du L., Fu S., Wan D., Bao Y., Feng Q., Grasso S., Hu C. (2022). Coatings.

[cit30] Zheng Z., Wu W., Yang T., Wang E., Du Z., Hou X., Liang T., Wang H. (2021). J. Adv. Ceram..

[cit31] Patil S. A., Marichev K. O., Patil S. A., Bugarin A. (2023). Surf. Interfaces.

[cit32] Cheng X., Cai J., Liu P., Chen T., Chen B., Gong D. (2024). Small.

[cit33] Zheng Z., Wu W., Yang T., Wang E., Du Z., Hou X., Liang T., Wang H. (2021). J. Adv. Ceram..

[cit34] Zhang L., Ding X., Lin D., Feng Y., Fu H., Xiao G., Xu P., Li Q. (2025). Composites, Part B.

[cit35] Mehdi S. M. Z., Ghulam Abbas H., Ali M., Rizvi S. B. H., Choi S. R., Goak J. C., Seo Y., Kumar S., Lee N. (2025). Energy Environ. Mater..

[cit36] Liu J., Liu Z., Zhang H.-B., Chen W., Zhao Z., Wang Q.-W., Yu Z.-Z., Liu J., Liu Z. H., Zhang B., Chen W., Zhao Z., Wang Q.-W., Yu Z.-Z. (2020). Adv. Electron. Mater..

[cit37] Ling Z., Ren C. E., Zhao M. Q., Yang J., Giammarco J. M., Qiu J., Barsoum M. W., Gogotsi Y. (2014). Proc. Natl. Acad. Sci. U. S. A..

[cit38] Sergiienko S. A., Lopes D. V., Constantinescu G., Ferro M. C., Shcherban N. D., Tursunov O. B., Shkepu V. I., Pazniak H., Tabachkova N. Y., Castellón E. R., Frade J. R., Kovalevsky A. V. (2021). Int. J. Hydrogen Energy.

[cit39] Sergiienko S. A., Lajaunie L., Rodríguez-Castellón E., Constantinescu G., Lopes D. V., Shcherban N. D., Calvino J. J., Labrincha J. A., Sofer Z., Kovalevsky A. V. (2024). RSC Adv..

[cit40] Wang W., Zhai H., Chen L., Huang Z., Bei G., Greil P. (2016). Mater. Sci. Eng., A.

[cit41] Scandura G., Kumari P., Palmisano G., Karanikolos G. N., Orwa J., Dumée L. F. (2023). Ind. Eng. Chem. Res..

[cit42] Juarez T., Biener J., Weissmüller J., Hodge A. M. (2017). Adv. Eng. Mater..

[cit43] Qi Z., Zhao C., Wang X., Lin J., Shao W., Zhang Z., Bian X. (2009). J. Phys. Chem. C.

[cit44] Bai Q., Zhang C., Tan F., Zhang Z. (2022). Intermetallics.

[cit45] Aburada T., Fitz-Gerald J. M., Scully J. R. (2011). Corros. Sci..

[cit46] Dai H., Shi S., Yang L., Guo C., Chen X. (2021). Corros. Rev..

[cit47] Dan Z., Qin F., Sugawara Y., Muto I., Hara N. (2012). Intermetallics.

[cit48] Young D. J., Wainwright M. S., Anderson R. B. (1980). J. Catal..

[cit49] Yu L., Jiang Y., He Y., Liu C. T. (2015). J. Alloys Compd..

[cit50] Cossar E., Houache M. S. E., Zhang Z., Baranova E. A. (2020). J. Electroanal. Chem..

[cit51] Handoko A. D., Fredrickson K. D., Anasori B., Convey K. W., Johnson L. R., Gogotsi Y., Vojvodic A., Seh Z. W. (2018). ACS Appl. Energy Mater..

[cit52] Yang L., Zheng W., Zhang P., Chen J., Tian W. B., Zhang Y. M., Sun Z. M. (2018). J. Electroanal. Chem..

[cit53] Wang H., Zhang J., Wu Y., Huang H., Jiang Q. (2018). J. Phys. Chem. Solids.

[cit54] Lukatskaya M. R., Kota S., Lin Z., Zhao M. Q., Shpigel N., Levi M. D., Halim J., Taberna P. L., Barsoum M. W., Simon P., Gogotsi Y. (2017). Nat. Energy.

[cit55] Xia Q. X., Fu J., Yun J. M., Mane R. S., Kim K. H. (2017). RSC Adv..

[cit56] de Oro Calderon R. , Gierl-Mayer C. and Danninger H., Encyclopedia of Materials: Metals and Alloys, 2021, pp. 481–492

[cit57] Gonzalez-Julian J. (2021). J. Am. Ceram. Soc..

[cit58] Magnuson M., Mattesini M. (2017). Thin Solid Films.

[cit59] Naslund L. Å., Persson P. O. Å., Rosen J. (2020). J. Phys. Chem. C.

[cit60] Ohtsu N., Oku M., Shishido T., Wagatsuma K. (2007). Appl. Surf. Sci..

[cit61] Zhou A., Wang C. A., Ge Z., Wu L. (2001). J. Mater. Sci. Lett..

[cit62] Biswas A., Roy S. K., Gurumurthy K. R., Prabhu N., Banerjee S. (2002). Acta Mater..

[cit63] Tzenov N. V., Barsoum M. W. (2000). J. Am. Ceram. Soc..

[cit64] Peng C., Wang C. A., Song Y., Huang Y. (2006). Mater. Sci. Eng., A.

[cit65] Massalski T. B. (1980). Bull. Alloy Phase Diagrams.

[cit66] Liu Y., Bailey P., Noakes T. C. Q., Thompson G. E., Skeleton P., Alexander M. R. (2004). Surf. Interface Anal..

[cit67] Su Y. R., Wu T. H., Cheng I. C. (2021). J. Phys. Chem. Solids.

[cit68] Zobac O., Kroupa A., Zemanova A., Richter K. W. (2019). Metall. Mater. Trans. A.

[cit69] Wang P. Y., Li H. J., Qi L. H., Zeng X. H., Zuo H. S. (2011). Prog. Nat. Sci.:Mater. Int..

[cit70] Galvin T., Hyatt N. C., Rainforth W. M., Reaney I. M., Shepherd D. (2018). J. Eur. Ceram. Soc..

[cit71] Qiu C., Metselaar R. (1994). J. Alloys Compd..

[cit72] Pietzka M. A., Schuster J. C. (1994). J. Phase Equilib..

[cit73] Xu L., Zhu D., Grasso S., Suzuki T. S., Kasahara A., Tosa M., nam Kim B., Sakka Y., Zhu M., Hu C. (2017). J. Adv. Ceram..

[cit74] Ovodok E. A., Ivanovskaya M. I., Poznyak S. K., Maltanova A. M., Azarko I. I., Micusik M., Omastava M., Aniskevich A. (2023). Thin Solid Films.

[cit75] Lu Q., Hutchings G. S., Yu W., Zhou Y., Forest R. V., Tao R., Rosen J., Yonemoto B. T., Cao Z., Zheng H., Xiao J. Q., Jiao F., Chen J. G. (2015). Nat. Commun..

[cit76] Liu L., Zhao Q., Liu R., Zhu L. (2019). Appl. Catal., B.

[cit77] Bao H., Qiu Y., Peng X., ao Wang J., Mi Y., Zhao S., Liu X., Liu Y., Cao R., Zhuo L., Ren J., Sun J., Luo J., Sun X. (2021). Nat. Commun..

[cit78] Bai Y., Liu C., Chen T., Li W., Zheng S., Pi Y., Luo Y., Pang H., Bai Y., Liu C., Chen T., Li W., Zheng S., Pi Y., Pang H., Luo Y. (2021). Angew. Chem., Int. Ed..

[cit79] Grujicic D., Pesic B. (2002). Electrochim. Acta.

[cit80] Beverskog B., Puigdomenech I. (1997). J. Electrochem. Soc..

[cit81] Norga G. J., Platero M., Black K. A., Reddy A. J., Michel J., Kimerling L. C. (1997). J. Electrochem. Soc..

[cit82] Schultz T., Frey N. C., Hantanasirisakul K., Park S., May S. J., Shenoy V. B., Gogotsi Y., Koch N. (2019). Chem. Mater..

[cit83] Zou G., Zhang Z., Guo J., Liu B., Zhang Q., Fernandez C., Peng Q. (2016). ACS Appl. Mater. Interfaces.

[cit84] Ghidiu M., Lukatskaya M. R., Zhao M. Q., Gogotsi Y., Barsoum M. W. (2014). Nature.

[cit85] Peera S. G., Koutavarapu R., Chao L., Singh L., Murugadoss G., Rajeshkhanna G. (2022). Micromachines.

[cit86] Lukatskaya M. R., Mashtalir O., Ren C. E., Dall'Agnese Y., Rozier P., Taberna P. L., Naguib M., Simon P., Barsoum M. W., Gogotsi Y. (2013). Science.

[cit87] Kong W., Deng J., Li L. (2022). J. Mater. Chem. A.

[cit88] Giri S. D., Sarkar A. (2016). J. Electrochem. Soc..

[cit89] Wan Y., Zhang Y., Wang X., Wang Q. (2013). Electrochem. Commun..

[cit90] Ferreira E. B., Jerkiewicz G. (2021). Electrocatalysis.

[cit91] Baranova E. A., Cally A., Allagui A., Ntais S., Wüthrich R. (2013). C. R. Chim..

[cit92] Luo J., Fang C., Jin C., Yuan H., Sheng O., Fang R., Zhang W., Huang H., Gan Y., Xia Y., Liang C., Zhang J., Li W., Tao X. (2018). J. Mater. Chem. A.

[cit93] Sundarraj S., Vadivel N., Murthy A. P., Theerthagiri J., Choi M. Y. (2024). Small.

[cit94] Shinagawa T., Garcia-Esparza A. T., Takanabe K. (2015). Sci. Rep..

[cit95] Magne D., Mauchamp V., Célérier S., Chartier P., Cabioc’h T. (2016). Phys. Chem. Chem. Phys..

[cit96] Solmaz R., Döner A., Kardaş G. (2008). Electrochem. Commun..

[cit97] Wang P., Xu D., Niu J. (2016). Appl. Phys. A: Mater. Sci. Process..

[cit98] X-ray Photoelectron Spectroscopy (XPS) Reference Pages: Copper, https://www.xpsfitting.com/2012/01/copper.html, accessed 9 April 2025

[cit99] Hsieh H., Chang Y., Pong W., Pieh J., Tseng P., Sham T., Coulthard I., Naftel S. (1998). Phys. Rev. B: Condens. Matter Mater. Phys..

[cit100] Ede S. R., Anantharaj S., Kumaran K. T., Mishra S., Kundu S. (2017). RSC Adv..

[cit101] Tang F., Wang L., Dessie Walle M., Mustapha A., Liu Y. N. (2020). J. Catal..

[cit102] Wang Z., Shen S., Wang J., Zhong W. (2024). Chem.–Eur. J..

