# Supplementary material for: Engineering MXene/metal composites from MAX phase/metal–Al precursors for high-performance energy conversion and storage
Source: RSC Adv. 2025 Nov 10;15(51):43505–22. doi: 10.1039/d5ra07113e (PMC12598392; doi:10.1039/d5ra07113e)

This journal is © The Royal Society of Chemistry 2025

Supplementary Information

Data for this article are available at Zenodo repository at https://zenodo.org/records/15280748

Table S1 Denominations and initial composition of the samples.

Sample denomination after sintering	Initial composition and molar ratios					Temperature, ° C	Time, min.
	Ni	Cu	Ti	Al	C		
S1	0.5		2	4.5	1	800	1
S2	1		2	4.5	1	800	1
S3	0.25	0.25	2	4.5	1	1100	30
S4	0.25	0.25	2	4.5	1	1200	30
S5	0.25	0.25	2	4.5	1	1450	30
S6		1.5	2	4.5	1	1000	240
S7		1.5	2	5	1	1000	240
S8		1.5	2	5.5	1	1000	240

Fig. S1 XRD data of the composites (Table S1): samples S1, S2, sintered at 800° C for 1 min. (A), samples S3, S4, S5 sintered for 30 min. at different temperatures 1100° C, 1200° C and 1450° C respectively (B), samples S6, S7, S8 sintered with different Al content at 1000° C for 240 min. (C), MXene/Cu composites samples 9b, 7b (D).

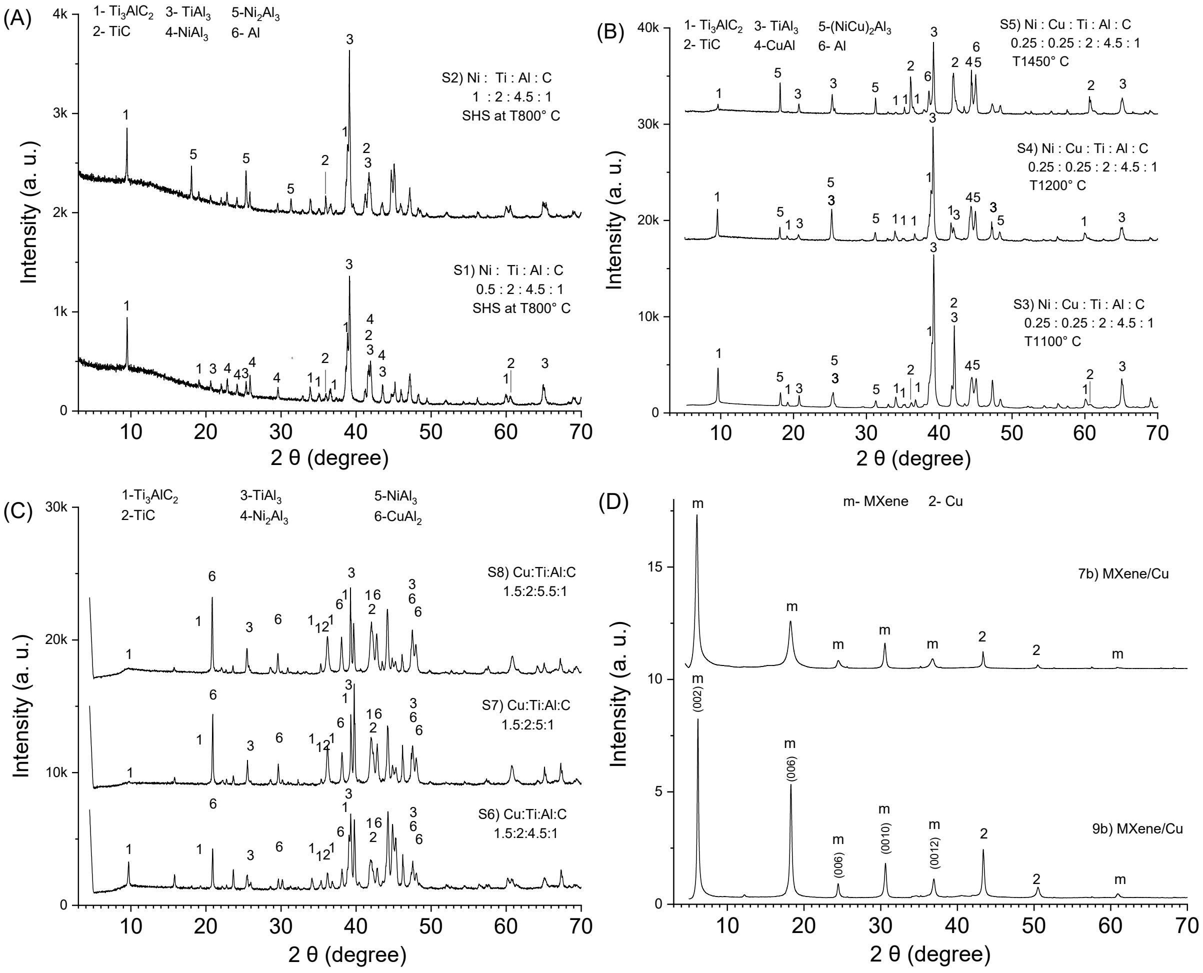

Fig. S2 Images of the composite: sample 10a (MAX phase/Cu) after etching in HCl solution (A, B).

(A)
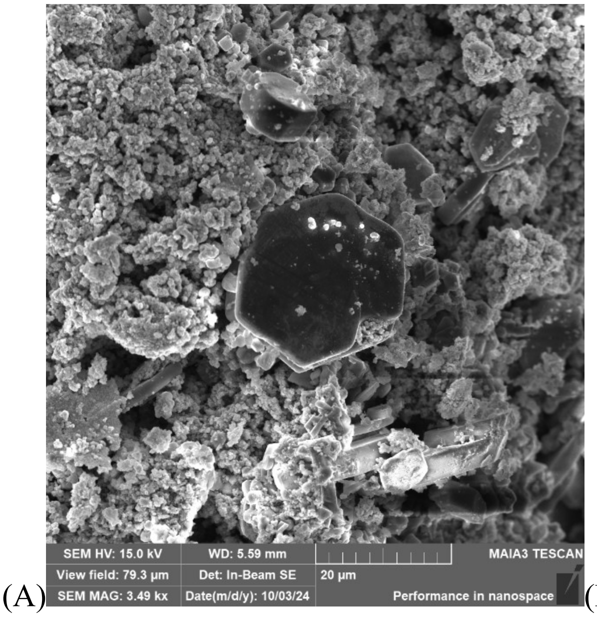

(B)
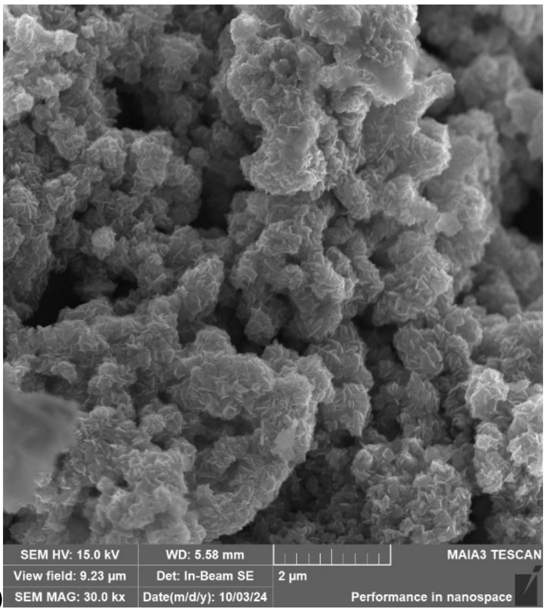

Fig. S3 Spectrum (A) and SEM/EDS mapping images of the interface of MXene/Cu sample 9b showing the distribution of C (C), O (D), F (E), Al (F), Ti (G), Cu (H), in the surface layer.

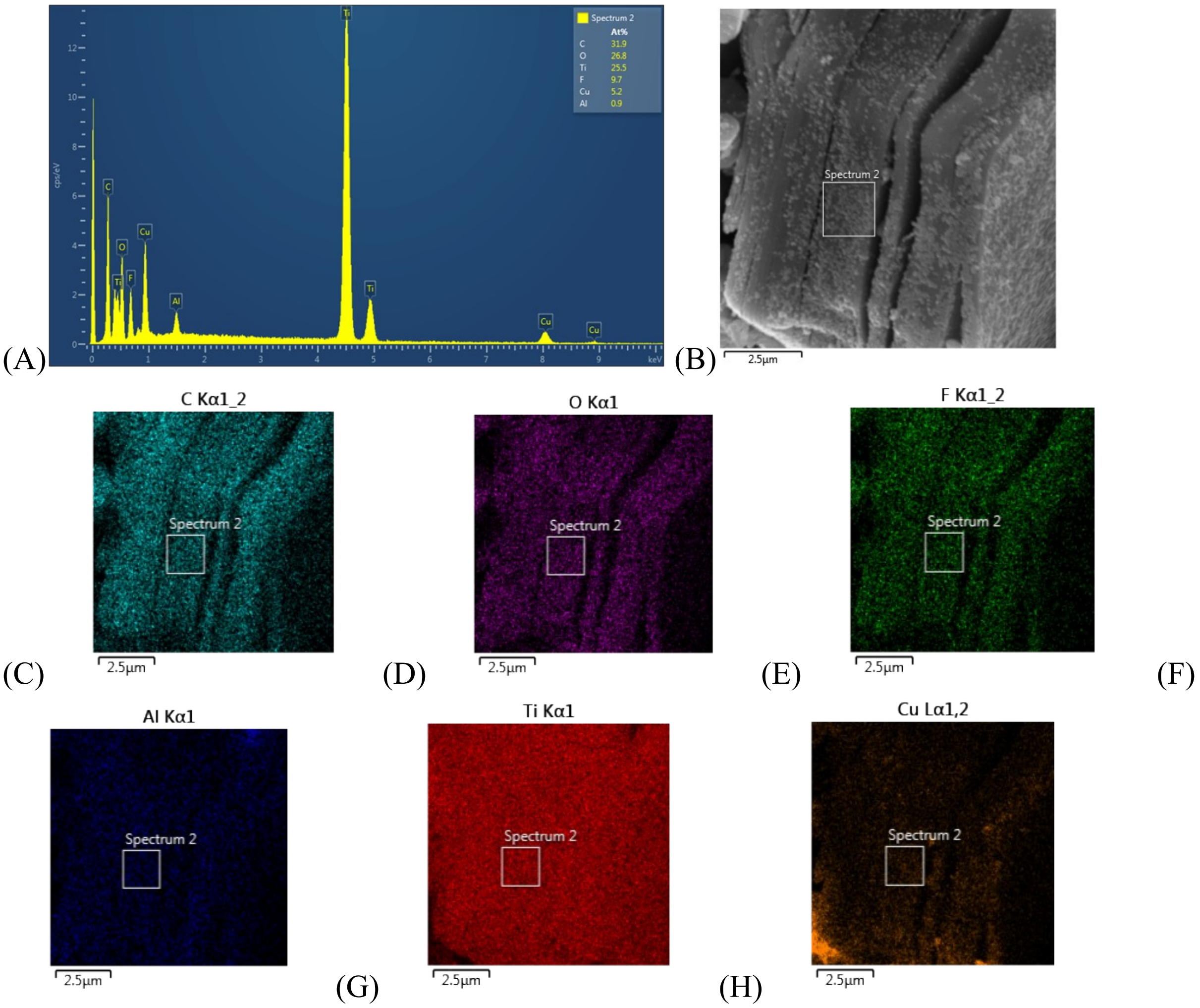

Fig. S4 CV curves at different scan rates of the sample 7b (A), 4b (B).

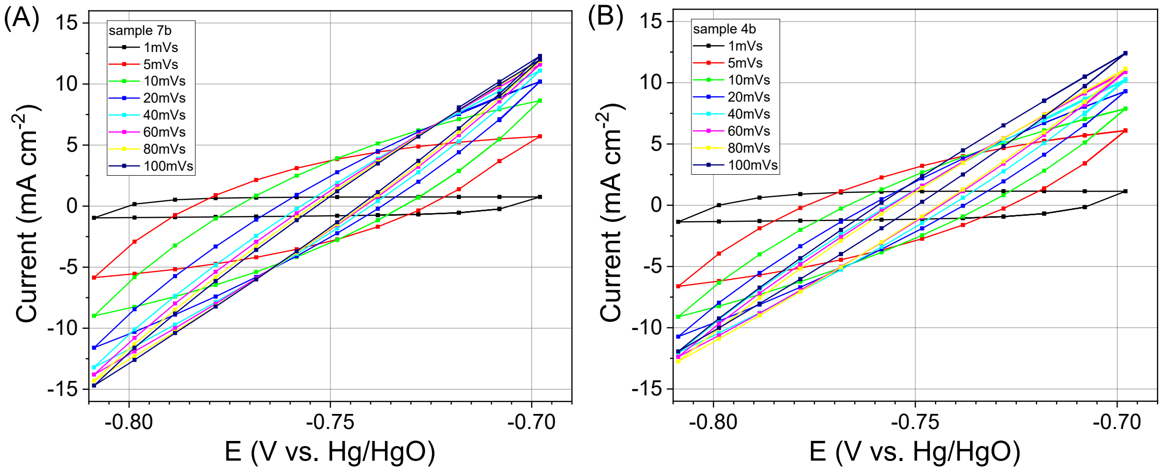

Fig. S5 CV curves at different scan rates of the sample 8b (A), 5b (B), 10b (C).

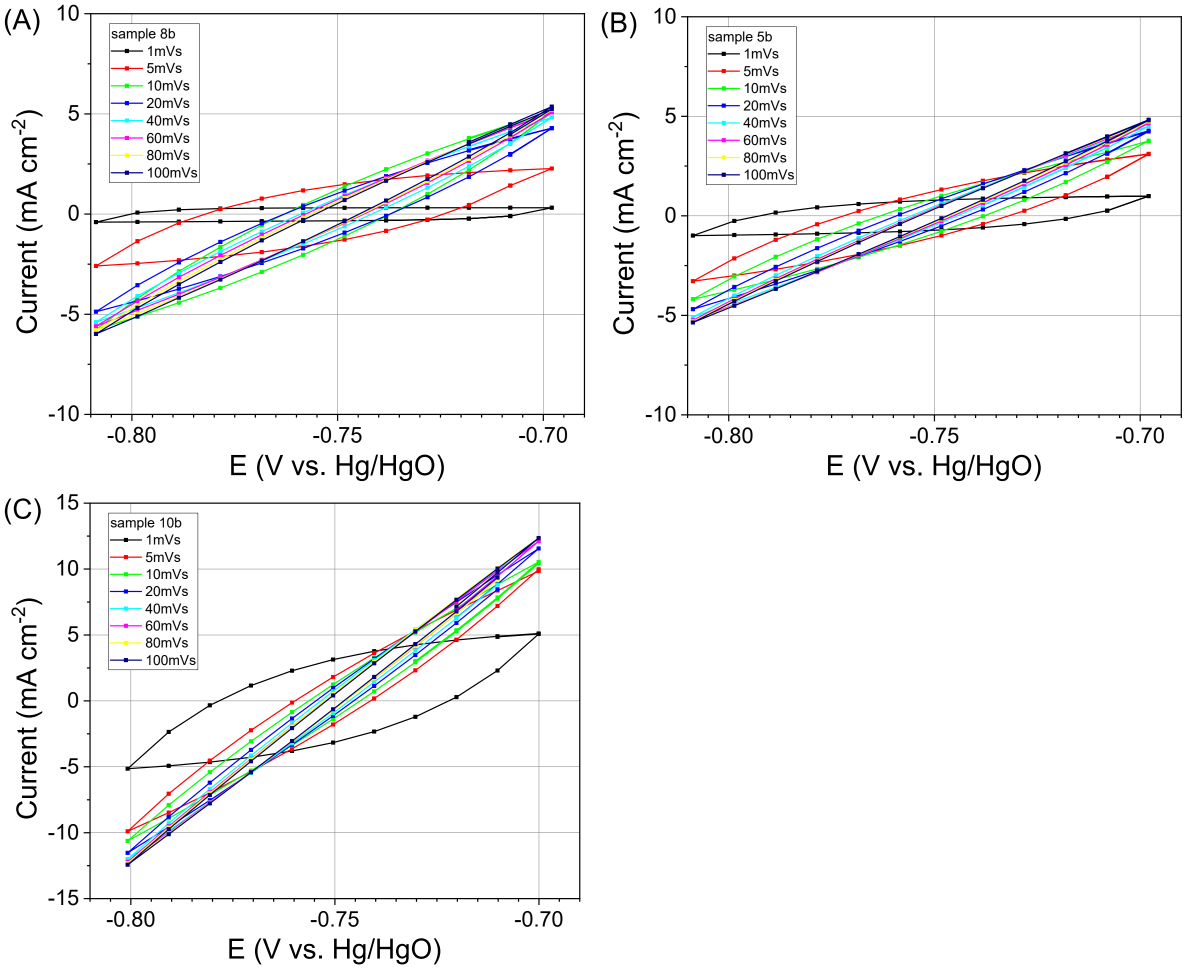

Supplement: RA-015-D5RA07113E-s001 [file RA-015-D5RA07113E-s001.pdf]
